# EnDisease: a manually curated database for enhancer-disease associations

**DOI:** 10.1093/database/baz020

**Published:** 2019-02-21

**Authors:** Wanwen Zeng, Xu Min, Rui Jiang

**Affiliations:** 1 MOE Key Laboratory of Bioinformatics; Bioinformatics Division and Center for Synthetic & Systems Biology, Department of Automation, Tsinghua University, Beijing, China; 2 Department of Computer Science and Technology; Institute for Artificial Intelligence, Bioinformatics Division, BNRist, Tsinghua University, Beijing, China

## Abstract

Genome-wide association studies have successfully identified thousands of genomic loci potentially associated with hundreds of complex traits in the past decade. Nevertheless, the fact that more than 90% of such disease-associated variants lie in non-coding DNA with unknown functional implications has been appealing for advanced analysis of plenty of genetic variants. Toward this goal, recent studies focusing on individual non-coding variants have revealed that complex diseases are often the consequences of erroneous interactions between enhancers and their target genes. However, such enhancer-disease associations are dispersed in a variety of independent studies, and thus far it is still difficult to carry out comprehensive downstream analysis with these experimentally supported enhancer-disease associations. To fill in this gap, we collected experimentally supported associations between complex diseases and enhancers and then developed a manually curated database called EnDisease (http://bioinfo.au.tsinghua.edu.cn/endisease/). Concretely, EnDisease documents 535 associations between 133 diseases and 454 enhancers, extracted from 199 articles. Moreover, after annotating these enhancers using 649 human and 115 mouse DNase-seq experiments, we find that cancer-related enhancers tend to be open across a large number of cell types. This database provides a user-friendly interface for browsing and searching, and it also allows users to download data freely. EnDisease has the potential to become a helpful and important resource for researchers who aim to understand the molecular mechanisms of enhancers involved in complex diseases.

## Introduction

With the development of genome-wide association studies (GWAS), the number of known disease-associated variants is booming and continues to expand (1, 2). There is no doubt that such fruitful resources could provide unprecedented opportunities for dissecting the genetics of complex diseases, thereby boosting the prevention, diagnosis and treatment of human diseases (3). However, the overall outcome of GWAS is currently unsatisfactory when considering the following three challenges. First, a statistically significant locus can often explain only a limited proportion of disease risk, leading to the missing heritability problem (4). Second, the prevalent existence of correlations between markers, also called linkage disequilibrium, makes precise identification of causal markers difficult (5). Third, most variants identified in GWAS lie in non-coding regions of the human genome with unknown effects (6). Due to our limited understanding of sophisticated non-coding regulatory relationships, it is unclear how these sequence variants affect gene expression and how they cause diseases. Therefore, one of the primary problems for researchers is to uncover the precise molecular mechanisms behind these variants.

Non-coding DNA sequences, including non-coding RNAs, enhancers, promoters, insulators and many other elements (7), are components of DNA that do not encode proteins. These non-coding elements fulfill a wide variety of crucial biological roles involving regulatory and signaling functions (1). Particularly, enhancer is one of the important non-coding elements that has a central role in controlling gene expression (8). They often function at a distance by forming chromatin loops to bring the enhancer and target gene into proximity, in a cell type-specific manner. Recent studies have uncovered that non-coding single nucleotide polymorphisms (SNPs) associated with risk for numerous complex diseases are enriched in cell type-specific enhancers (9, 10). Specifically, more than 1 million disease-associated SNPs have been documented to accumulate in enhancers (11, 12), yielding the emergence of a coherent picture regarding whether diseases are often the consequence of inaccurate interactions between enhancers and their target genes (13).

Recently, studies that focus on individual non-coding genetic variants associated with specific diseases demonstrated that these variants in enhancers can cause diseases through a diverse range of molecular mechanisms (14, 15). First, enhancers can be deleted, weakened or strengthened (16). All of these situations may lead to transcriptional dysregulation of the original target gene. Particularly, amplifications of enhancers are a common mechanism for upregulating the expression of cancer driver genes. For instance, a GWAS-identified risk variant associated with neuroblastoma is found to form an extra transcription factor binding site in an enhancer that leads to overexpression of the LMO1 oncogene (17). Another example is that mutations in the distal ZRS enhancer may recruit new transcription factors and drive Shh expression in ectopic sites of the limb bud (18). Second, a new enhancer or promoter can be introduced and upregulate the target gene. In leukemia, some mutations introduce new transcription factor binding motifs and result in a new enhancer, which causes overexpression of the nearby TAL1 oncogene (19). As another example, a GWAS-identified risk variant, which is associated with both plasma low-density lipoprotein cholesterol and myocardial infarction, is shown to create a transcription factor binding site that enhances expression of nearby SORT1, a gene relevant for lipoprotein metabolism (20). Third, a new target gene can be activated in an unexpected way. Rearrangements, such as inversions or translocations, can place an enhancer in a new genomic context where the enhancer can activate a new target gene. A classic example is the t(8;14) translocation in Burkitt lymphoma that moves the enhancer of immunoglobulin heavy chain toward the MYC gene (21, 22). This phenomenon makes the enhancer regulate the expression of MYC in an unexpected way, also known as ‘enhancer hijacking’. As an additional example in cancers, medulloblastoma is frequently caused by rearrangements that place the GFI1 gene family under the control of super enhancers (23).

Considering the above diverse functional implications of genetic variants in enhancers, we hope to systematically assign functional annotations to the ever-expanding collection of enhancers associated with diseases and further find out the diverse patterns of these genetic variants in enhancers leading to different diseases. To characterize the status of experimentally validated disease-related enhancers in different cell types and species, we develop EnDisease, a manually curated database that records experimentally supported enhancer-disease associations with comprehensive chromatin accessibility annotations. Specifically, EnDisease documents 515 experimentally supported human/mouse enhancer-disease associations and annotates the enhancers with 649 human and 115 mouse DNase-seq experiments (24) in different cell types. Besides, EnDisease also collects 20 enhancer-disease associations in nine other species for the sake of future cross-species studies. Through the analysis of the chromatin accessibility profiles of disease-related enhancers in different cell types, we believe that there might be different patterns of regulation for enhancers related to complex diseases, especially cancers. In general, EnDisease can serve as a useful resource for researchers who want to further investigate mechanisms between enhancers and diseases.

**Table 1:** Statistics for enhancer-disease associations in the EnDisease database. Each row represents a species. The enhancers column indicates the number of enhancers involved in this species, the diseases column indicates the number of diseases involved in this species and enhancer-disease associations columns indicates the number of enhancer-disease association in this species. The associations we collected are mainly from human. The last line represents the total number of enhancers, diseases and enhancer-disease associations across species. Note that the total number of diseases is not a direct summation since some diseases are studied across different species

Species	Enhancers	Diseases	Enhancer-disease associations
*Homo sapiens*	413	125	483
*Mus musculus*	21	13	32
Others	20	4	20
Total	454	133	535

## Results

### Database content

The current version of EnDisease contains enhancer-disease associations that are obtained from literature review. After systematically reviewing 199 published articles, a total of 535 entries representing 454 enhancers and 133 diseases are manually collected. The number of enhancer-associated entries for each species is listed in Table 1. Each entry involves detailed information (Methods), including disease information (i.e. disease name and associated mutation information, such as GWAS risk SNPs), enhancer information (i.e. chromosome, strand, start site, end site, associated genes, species, reference genome, cell type) and publication information (i.e. PubMed ID). Note that all the enhancer coordinates have been lifted over to the newest version of genome for each species (Methods), e.g. hg38 for *Homo sapiens* and mm10 for *Mus musculus*, as listed in Supplementary Table 1. To distinguish EnDisease from other databases, we further collect 649 DNase-seq open chromatin experiments for human and 115 DNase-seq experiments for mouse to annotate the epigenomic states of our enhancers. For each experiment, we first run the ENCODE (25) processing pipeline and then annotate the openness scores for each enhancer (Methods). We double check all entries according to inclusion criteria (Methods) and discard uncertain ones that lack sufficient details. EnDisease provides a hyperlink to the Online Mendelian Inheritance in Man (OMIM) database (26) for readers to get more information about a disease, a hyperlink to UCSC Genome Browser (27) to let readers gain more concrete knowledge of an enhancer, and a hyperlink to the literature on PubMed for readers to learn the specific methods and results behind an enhancer-disease association (Methods).

**Figure 1:**
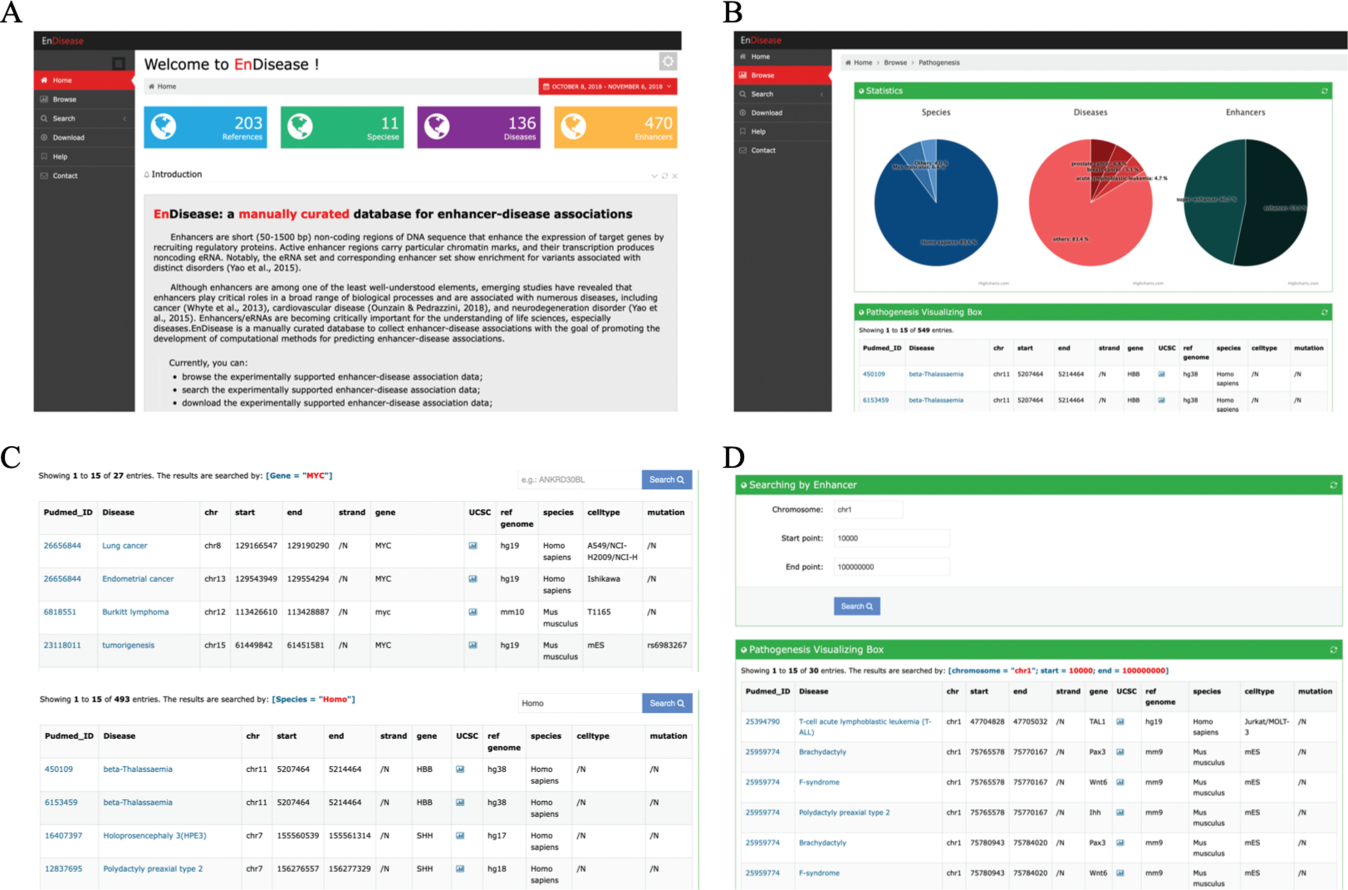
Overview of EnDisease Website. (A) Overview of EnDisease. (B) Figure 1. `Browse’ page of EnDisease. (C) Example of `Search’ page by genes and species. (D) Example of `Search’ page by enhancers.

### User interface

EnDisease offers a user-friendly web interface to promote access to the database. The web interface provides three main components: (i) a browsing service for catching a glimpse of all data, (ii) a search service for retrieving specific enhancer-disease associations and (iii) a download service for downloading a local copy of this database and other annotation files. To help new users make better use of the services, EnDisease also provides a user guide in the help page (Figure 1a).

#### Browse page

In the ‘Browse’ page, users can browse all data and get an overall impression of EnDisease database (Figure 1b). The database can be browsed for each species by clicking the ‘Browse’ tab on the left-side navigation menu, and the results will be displayed in a large table. In this table, each row is an enhancer-disease association, and each column contains a type of association-related information, e.g. disease name, a hyperlink to the OMIM database, PubMed ID, a hyperlink to PubMed website, a hyperlink to download openness scores, enhancer genomic loci (chromosome, start site, end site, strand), a hyperlink to UCSC website, associated genes, associated mutations, species and cell type. Each page displays 15 entries, and users can view remaining entries using pagination features on the bottom right of the table. The detailed information of each entry on the ‘Browse’ page is listed in Supplementary Table 2.

#### Search page

In the ‘Search’ page, EnDisease allows users to search by enhancers, diseases, cell types and species names. The search results are organized in the same way as the ‘Browse’ page. Users can perform fuzzy search by species using the ‘species’ search box, by diseases using the ‘diseases’ search box and by cell types using the ‘cell types’ search box (Figure 1c). For example, we could enter ‘*Homo sapiens*’ in the species search box to display all human enhancer-disease associations.

As for searching by enhancers, EnDisease also supports fuzzy search function (Figure 1d). Users can input chromosome, enhancer start site and enhancer end site, and then all possible entries with enhancers located in this region are displayed. For example, users could enter a tuple of (chr1, 100000, 1000000) to query entries with enhancers inside this region, and then the qualified enhancer-disease associations are displayed in a table.

#### Download page

In the ‘Download’ page, EnDisease allows users to download all EnDisease entries in a single file. For each DNase-seq experiment, EnDisease provides detailed information such as ENCODE access ID and cell line, in the ‘Download’ page. Besides, it also provides a complete annotation matrix with detailed row names and column names. These experimental annotations of enhancers can help researchers for downstream analysis.

#### Help page

Last but not least, EnDisease provides a detailed database manual in the ‘Help’ page. We offer a step-by-step tutorial for EnDisease usage and list a number of frequently asked questions and their answers in the ‘Help’ page.

### Statistical description of EnDisease

In this section, we will make a statistical description of EnDisease from multiple aspects.

First, the most frequent three disease types occurring in EnDisease are prostate cancer (6.74%), breast cancer (5.10%) and acute lymphoblastic leukemia (4.74%). These top-ranked diseases are related to tumors, which is in line with the fact that super-enhancers play important roles in the misregulation of gene expression in cancers (28, 29).

**Figure 2:**
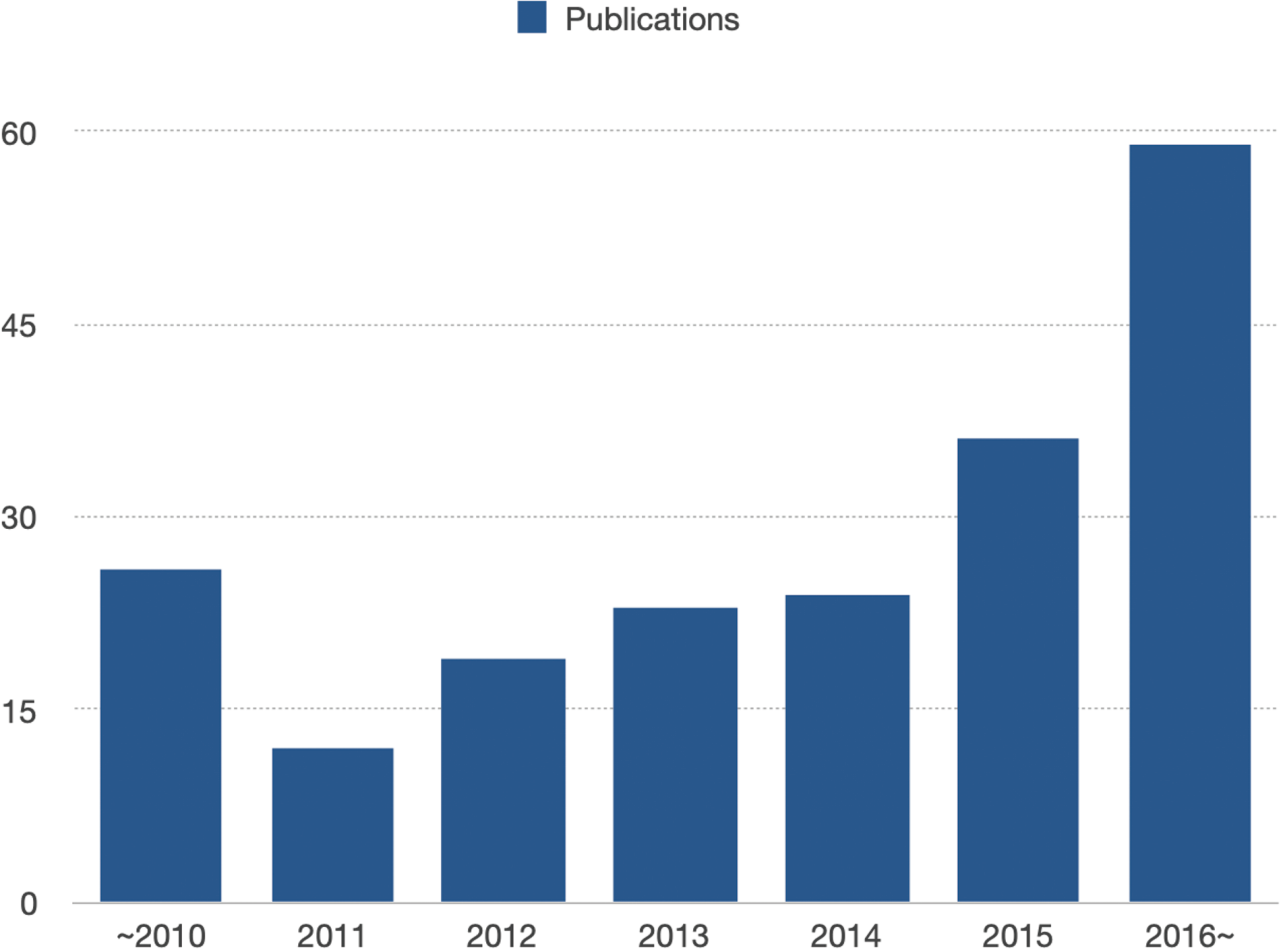
The number of entries in recent years. The number of articles regarding enhancer-disease associations keeps increasing year by year.

**Figure 3:**
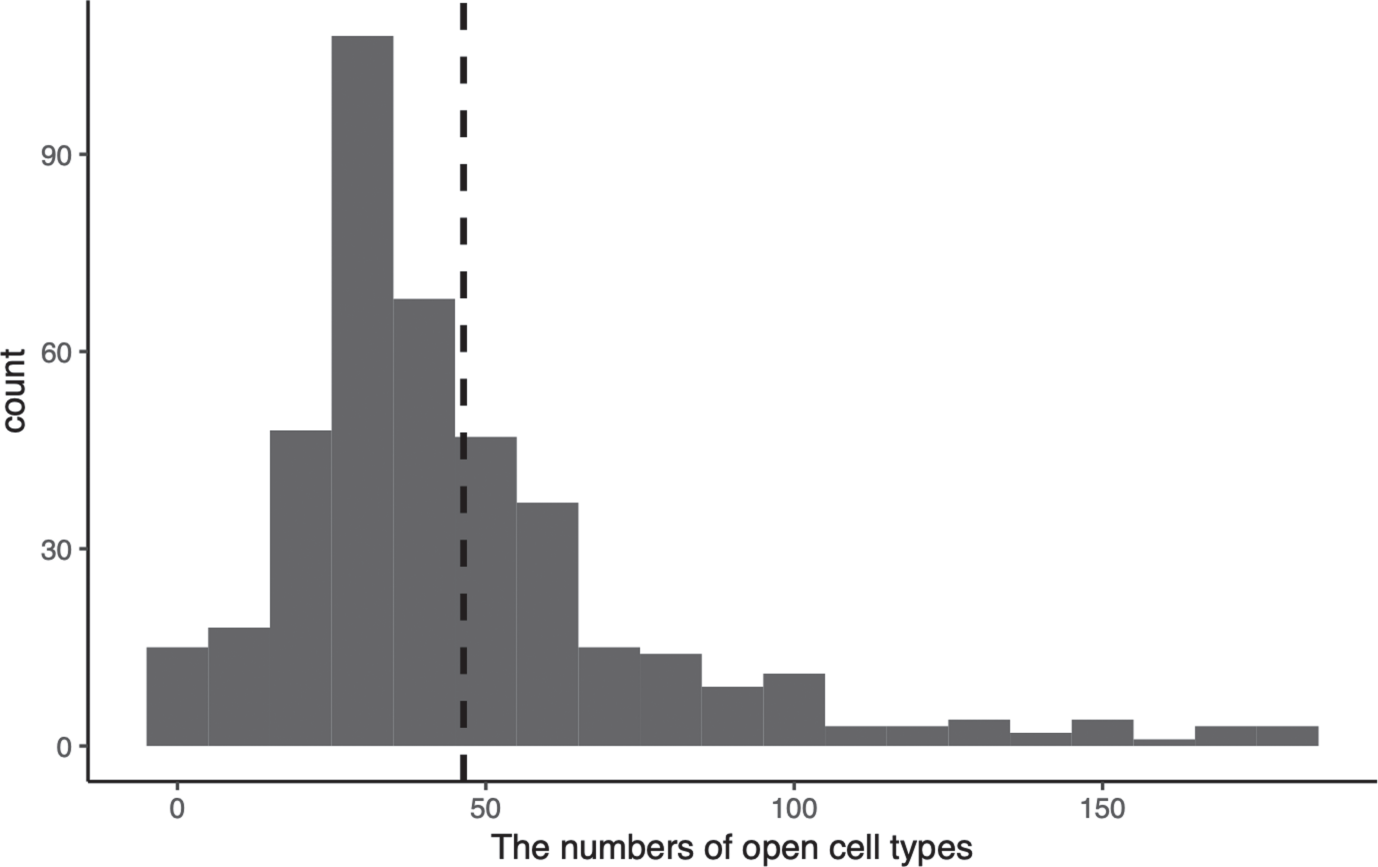
Distribution of the numbers of open cell types of disease-enhancers. The x-axis indicates the numbers of open cell types, and the y-axis indicates the count of collected enhancers. The dash line represents the mean open cell types for all enhancers.

Second, EnDisease collects chromatin accessibility DNase-seq experiments in different cell types: 649 human DNase-seq experiments in 184 cell types and 115 mouse DNase-seq experiments in 30 cell types. After running ENCODE (25) DNase-seq pipeline for each experiment, we annotate each disease-related enhancer with the obtained openness scores (Methods). Among these experiments, the most frequent three tissues for human are kidney (13.25%), muscle (9.40%) and brain (6.93%), while the top three tissues for mouse are stem cells (11.55%), brain (11.55%) and blood (8.04%). The detailed information for each experiment is provided in Supplementary Table 3. We expect that researchers can gain a better understanding of disease mechanism for these enhancers with comprehensive openness scores in different cell types.

Third, by analyzing the number of disease-associated enhancers articles in EnDisease, we find that such articles keep increasing year by year (Figure 2), suggesting that research on enhancer-disease associations remains one of the hottest topics in the field of complex diseases in this decade. A recent review article defines a roadmap called ‘from disease-associated variants to molecular mechanisms causing disease’ (15) and provides a uniform and normative method to interpret how disease-associated variants lead to diseases. Following this roadmap, it is foreseeable that more high-quality articles will be published with more detailed information for enhancer-disease associations, and we will integrate them into our EnDisease database.

In summary, with the aim to promote the transition from anecdotal descriptions of risk variants to discovery of molecular mechanisms underlying diseases, we will continue to pay attention to enhancer-disease studies, collect, annotate and record these associations, to make EnDisease more conducive to downstream analysis.

### Analysis of disease-enhancers across chromatin accessibility annotations in different cell types

We annotate each human enhancer with 649 DNase-seq openness scores (Supplementary Table 4) and get an openness score matrix, where each row represents an enhancer and each column represents a cell type. If there are multiple experiments in a single cell type, we merge them by calculating the mean openness score. We define an enhancer as open in a specific cell type if the enhancer has an openness score larger than 2 in the cell type, define an enhancer as cell type-specific if the enhancer is open in less than 37 (20% of collected cell lines) cell types and define an enhancer as permissive if the enhancer is open in more than 110 (60% of collected cell lines) cell types.

We draw the distribution of the numbers of open cell types for all disease-enhancers in Figure 3, from which we find that 53.51% enhancers are cell type-specific, while 5.32% enhancers are permissive. Furthermore, among these permissive enhancers, 77.27% (17 out of 22) of them are related to cancers. These results from simple statistical analysis suggest that cancer-related enhancers are likely to be permissive. To further validate this conjecture, we perform a one-sided Wilcoxon rank sum test against the alternative hypothesis that cancer-related enhancers are open in more cell types than enhancers irrelevant to cancer. The small *P*-value ($4.33\times {10}^{-9}$) demonstrates that cancer-related enhancers indeed function in more cell types than non-cancer related enhancers.

One guess for this phenomenon is that these enhancers function in ubiquitous cell types to control the expression of oncogenes, and they may lead to cancers once they have been mutated in some specific cases (14, 15). In fact, although most enhancers drive cell type-specific gene expression, some enhancers might be ubiquitous and make some genes, such as house-keeping genes, express constantly in different experimental conditions. Zabidi *et al.* (39) used STARR-seq to screen the whole fly genome with different core promoters from either ubiquitously expressed housekeeping genes or developmentally regulated and cell type-specific genes (30). They found that promoter-proximal enhancers are mainly ubiquitously expressed and regulate promoters of housekeeping genes, while promoters of developmental genes are cell type-specific and required distally located enhancers.

With comprehensive epigenomic annotations across different cell types, we can distinguish permissive enhancers and cell type-specific enhancers, and we should continue to carry out in-depth studies on the regulation patterns of these enhancers for different diseases.

## Discussion

Researchers have been trying to decipher the precise molecular and cellular regulatory mechanisms involved in complex diseases. In recent years, emerging research work has focused on exploring potential mechanisms involving enhancers in diseases. Consequently, enhancer-disease associations are accumulating rapidly in this decade. However, most associations are dispersed in many independent studies. It becomes increasingly obvious that an integrated collection of enhancer-disease associations is critical for a complete understanding of mechanism underlying diseases. Thus, we develop EnDisease, a disease-specific database that provides a precious resource on enhancer-disease associations with diverse epigenomic annotations.

As a comparison, DiseaseEnhancer (31) is another new database that collects experimentally supported enhancers-diseases associations in human. For the source of candidate publications, DiseaseEnhancer uses some general keywords to select their candidates, while EnDisease uses detailed keyword combinations (using specific diseases in OMIM database as keywords) to select candidate articles. For inclusion criteria, DiseaseEnhancer collects both predicted and experimental enhancer-disease associations, while EnDisease collects only experimental validated enhancer-disease associations and experimental validated enhancer-pathogenesis genes associations. For example, in (32) the authors proposed a chromatin structure-based methods to predict recurrent non-coding mutations in cancer. They performed reporter assays on 10 of all 308 predicted mutations, and only four of these mutations caused a significant increase in luciferase activity. Nevertheless, DiseaseEnhancer still documents all the 308 enhancer-disease associations. For downstream analysis, we download comprehensive chromatin accessibility experiments and run ENCODE processing pipelines for these experiments to annotate our enhancers. By contrast, DiseaseEnhancer annotates detailed variants information, such as variants consequence. In summary, DiseaseEnhancer and EnDisease use different ways to collect enhancer-disease associations. Meanwhile, DiseaseEnhancer pays more attention to mutation information, while EnDisease puts more effort in annotating epigenomic status of these disease enhancers.

We plan to update our database regularly in the future. Most of the enhancer-disease associations are dispersed in many independent studies. It might take several years to discover candidate enhancers set and validate some of them using different biological experiments such as mouse experiment, and hence only a few articles (about 50) will be published per year. Besides, it will take us up to several months to perform the process of database maintenance. Therefore we expect to update EnDisease every 2 years.

In conclusion, recent studies have shed light on the mechanisms involved in the enhancer-disease associations. EnDisease not only provides a comprehensive database of experimentally supported enhancer-disease associations, but also offers a more global perspective on enhancer epigenomic profiles in different cell types. In the future, we will continue to manually collect newly validated enhancer-disease associations. In addition, we plan to integrate more sources such as functional annotations and provide additional tools such as an enhancer-disease association prediction tool. EnDisease will serve as a valuable resource for deciphering enhancer mechanisms and improving the diagnosis and treatment of complex diseases.

## Materials and methods

### Data preparation

#### Literature search and review

To ensure database quality, we referred to the steps used in the establishment of other manually curated databases (33–35). We followed three main steps in the data collection process: (i) defining candidate diseases (general and specific), (ii) searching for relevant articles and (iii) extracting useful information from the selected articles.

First, candidate diseases were selected using either general keywords (‘cancer’, ‘tumor’ and ‘disease’) or specific keywords from the disease vocabulary of the OMIM database, which contains diseases and disease-related genes information. We collected 8759 OMIM diseases terms in total, and used their disease titles, alternative titles and symbols recorded in OMIM as keywords for searching in PubMed. The candidate enhancers were hard to collect since there are no unified names or symbols for enhancers. Therefore we simply use ‘enhancer’ as a general keyword for fuzzy search.

Second, we used program scripts to automatically fetch all abstracts in the PubMed database with either two general keywords, such as [‘cancer’ and ‘enhancer’], [‘tumor’ and ‘enhancer’], [‘disease’ and ‘enhancer’] or a combination of a specific disease name and ‘enhancer’. For example, for OMIM #103100 we searched with keywords [‘ADIE PUPIL’ and ‘enhancer’], [‘ADIE SYNDROME’ and ‘enhancer’], [‘POORLY REACTING PUPILS’ and ‘enhancer’] in PubMed.

Third, we downloaded all the selected publications and available supplementary files that describe the associations between diseases and enhancers. We manually extracted experimentally supported enhancer-disease associations from these articles according to the following inclusion criteria.

#### Inclusion criteria

For enhancer-disease associations, we confirmed them as experimental validated using two criteria. First, the authors should validate whether the variation of the enhancer indeed causes disease by performing downstream biological experiments, rather than by prediction. There are different kinds of experiments: mouse experiments, which are the most intuitive to directly see the phenotype, and other indirect experiments, such as reporter assays detecting the effects of enhancers on the status of pathogenic genes (aberrant regulation). Second, the authors should provide the exact detailed information of the enhancer, including the chromosome, start site and end site. Note that, different articles might describe the same enhancer-disease associations in different ways (having different types of variations or affecting different genes), so we regarded them as different enhancer-disease associations.

For ‘enhancer-target gene associations’, the inclusion criterion is similar to that for enhancer-disease associations. The authors should perform experiments to validate enhancer-target gene associations. For example, the authors should perform chromosome conformation capture-based methods such as Hi-C (36), luciferase report assays (37) and etc. If the authors did not perform detailed experiments, we would not record the enhancer-target gene associations.

For ‘enhancer definitions’, we strictly followed enhancer locations in the original publications. The authors should provide detailed information of enhancer including the chromosome, start site, end site and reference genome. If the authors did not provide detailed information in the main manuscript or supplementary material, we would discard this enhancer-disease association.

#### Data annotations

After screening the data, we continued to annotate these selected associations. We provided three categories of information: (i) disease information, (ii) enhancer information and (iii) publication information.

‘Disease information’ includes disease name, associated mutation and a hyperlink to the disease term in OMIM database. For diseases in OMIM database, we could access the corresponding web pages in OMIM through the OMIM IDs. Users could learn detailed information, such as symptoms and disease genes, from the web pages.

‘Enhancer information’ includes species, reference genome, chromosome, start site, end site, strand, associated genes and hyperlink to UCSC genome browser. We extracted information in the publications and supplementary files. Enhancers usually regulate their target genes within topologically associating domains, which are the structural and functional units of chromosomes. Publications in EnDisease indicate that genetic variation can alter genomic interactions, resulting in transcriptional changes that finally lead to disease. Therefore, studying disease-associated variants in the context of the 3D organization of the genome can provide insights into the molecular mechanisms underlying diseases. For articles where experimentally verified enhancer-gene associations were available, gene information was assigned to enhancers based on their studies.

To distinguish EnDisease from other databases, we further collected 649 human and 115 mouse DNase-seq open chromatin experiments, and run the standard DNase-seq processing pipeline from ENCODE to annotate the epigenomic states of these enhancers. The steps are as follows: (i) preparation: bwa (40) indexing and hotspot mappability/blacklist preparation; (ii) alignment by bwa; (iii) merging and filtering of bams and quality control (QC) analysis; (iv) BAM evaluation script; (v) peak/hotspot calling by hotspot. Then we proposed a method to quantify the degree of chromatin accessibility (i.e. openness score) for an enhancer in an experiment, with the consideration that the score should be comparable across different experiments. Briefly, given an enhancer of length *L*, we treated this enhancer as foreground and denoted the count of reads in the enhancer region by *X*. To remove the sequencing depth effect, we chose a background region with length ${L}_0$ centered at this enhancer and denoted the count of reads in this background window by *Y*. We chose ${L}_0$=1 Mb as the length of background region (38). The openness score is then formally defined as the fold change of read numbers per base pair and can be simply calculated as

$$ o=\frac{\left(X+\delta \right)/L}{\left(Y+\delta \right)/{L}_0}, $$

where δ is a pseudocount (the default value of δ is 5 in our implementation).

‘Publication information’ includes PubMed ID and publication year and hyperlink to the PubMed website. Users could learn the journal name, author information, main ideas, methods and results from the PubMed website.

### Database implementation

#### Database construction

The current version of EnDisease was developed using MySQL (http://www.mysql.com) and was run on a Linux-based Apache server. We used PHP (http://www.php.net/) for server-side scripting. The interactive and responsive user interface was designed and built using Bootstrap (http://www.getbootstrap.com), a popular responsive development framework including HTML, CSS and JavaScript. The user interface is responsive, meaning that the web interface detects the user device and changes its structure and shape according to the device resolution in order to optimize the data view. This feature makes the interface compatible across a variety of devices and browsers with different screen resolutions. The database can be browsed and searched using a variety of devices including smartphones or tablets. Google Chrome and Safari web browsers are preferred for better user experience, while other current standard web browsers are also supported, including Internet Explorer. We aim to improve the accessibility and user interactivity of EnDisease by asking for user feedback through the contact page on our website.

#### External link construction

There are three kinds of external links in EnDisease: (i) PubMed website; (ii) OMIM website; (iii) UCSC website. For PubMed website, we extracted the PubMed ID, such as 6153459, for the entry and then appended it to the end of the link https://www.ncbi.nlm.nih.gov/pubmed/ as https://www.ncbi.nlm.nih.gov/pubmed/6153459/ to finish PubMed link construction; For OMIM website, we extracted the OMIM ID, such as 613985, for the entry and appended it to the end of the link https://www.omim.org/entry/ as https://www.omim.org/entry/613985 to finish OMIM link construction. To facilitate visualization of enhancers, we extracted the chromosome (chr11), start site (5155389), end site (5266609) and species (*H. sapiens*) for the entry and mapped the species to reference genome (hg38) using Supplementary Table 1. We put this information to the link http://genome.ucsc.edu/ cgi-bin/hgTracks?db=(reference_genome)&position=(chro mosome):(start_site)-(end_site) as http://genome.ucsc.edu/cgi-bin/hgTracks?db=hg38&position=chr11:5155389-5266609 to finish UCSC link construction.

#### Data storage

EnDisease contains 535 enhancer-disease associations. We do not maintain a disease table, an enhancer table, a species table and a publication table separately. Instead, we adopt a better solution, which is to use one big table in MySQL to store all the information, using PubMed ID, OMIM ID, chromosome, start site and end site as the primary key. With this big table, we could easily query different types of information without joining many tables, which is time efficient. Nevertheless, as the number of enhancer-disease associations increases, we might change our data storage format in the future to make it suitable for the expansion of the data scale.

#### Availability

The EnDisease database is freely available to the research community using the web link (http://bioinfo.au.tsinghua.edu.cn/endisease/). Users are not required to register or login to access any feature available in the database. In addition to the online service, we also provide a download service so that users could make use of all the data in EnDisease in a local version.

## Supplementary Material

Supplementary Data

## Notes

**Database URL:** http://bioinfo.au.tsinghua.edu.cn/endisease/
