# Supplementary material for: EnDisease: a manually curated database for enhancer-disease associations
Source: Database (Oxford). 2019 Feb 21;2019:baz020. doi: 10.1093/database/baz020 (PMC6382991; doi:10.1093/database/baz020)
Supplement: Supplementary Data [file baz020_supp.zip › supplementary_201901012.docx]

**Supplmentary - EnDisease: a manually curated database for enhancers-diseases associations**

**Wanwen Zeng^1^, Xu Min^2^, Rui Jiang^1,*^**

^1^ MOE Key Laboratory of Bioinformatics; Bioinformatics Division and Center for Synthetic & Systems Biology; Department of Automation, Tsinghua University, Beijing, 100084, China

^2^ Department of Computer Science and Technology; Institute for Artificial Intelligence; Tsinghua-Fuzhou Institute for Data Technology; Bioinformatics Division, BNRist, Tsinghua University, Beijing, 100084, China

^*^ Corresponding author

Email addresses:

W.W.Z : [zengww14@mails.tsinghua.edu.cn](mailto:zengww14@mails.tsinghua.edu.cn)

X.M : minx14@mails.tsinghua.edu.cn

R.J. : [ruijiang@tsinghua.edu.cn](mailto:ruijiang@tsinghua.edu.cn)

**Supplementary Table 1. Reference genome for each species.** We lifted over human and mouse genome coordinates to the newest version (hg38 for Homo sapiens and mm10 for Mus musculus) and kept the genome coordinates for other speices.

| Species | Reference genome |
| --- | --- |
| Homo sapiens | hg38 |
| Mus musculus | mm10 |
| Anolis carolinensis | anoCar1 |
| Bos taurus | bosTau4 |
| Drosophila melanogaster | dm6 |
| Gallus gallus | galGal3 |
| Ornithorhynchus anatinus | ornAna1 |
| Oryctolagus cuniculus | oryCun2 |
| Oryzias latipes | oryLat2 |
| Rattus norvegicus | rn6 |

**Supplementary Table 2. Detailed information of each entry.** Each row represents an attribute of each enhancer-disease association entry. There are mainly three types of information, including disease-related, enhancer-related, and publication-related information. Users can have basic understanding through these types of information.

| Column | Detailed information |
| --- | --- |
| disease | Disease name and a hyperlink to OMIM web page |
| mutation | The mutation information involved in enhancer-disease associations |
| chr | The chromatin of the enhancers |
| start | The start site of the enhancers |
| end | The end site of the enhancers |
| strand | The strand of the enhancers |
| gene | The enhancers’ target genes |
| UCSC | A hyperlink to UCSC genome browser to get detailed information of the enhancer regions |
| species | The species where enhancer-disease associations are validated |
| cell type | The cell type where enhancer-disease associations are validated |
| openness | A hyperlink to download openness scores for the enhancers |
| pudmed ID | PubMed ID of original literatures for the enhancer-disease associations and a hyperlink to the literatures in Pubmed |
