# Supplementary material for: EnDisease: a manually curated database for enhancer-disease associations
Source: Database (Oxford). 2019 Feb 21;2019:baz020. doi: 10.1093/database/baz020 (PMC6382991; doi:10.1093/database/baz020)
Supplement: Supplementary Data [file baz020_supp.zip › revision_response_20190102_final.docx]

Dear Editor,

We appreciate the editor for offering us the opportunity to revise the manuscript, and we thank the three reviewers for their careful evaluation of our manuscript. We would like to submit the revision of our manuscript titled “*EnDisease: a manually curated database for enhancers-diseases associations*”. In the revision, according to the comments of reviewers, we have made the following major changes to our original submission.

1) We updated the Introduction Section to claim the novelty and advantages of EnDisease. We compared EnDisease and DiseaseEnhancer from the perspective of methods and results in the Discussion Section.
2) We updated Section 2.3 to “Statistical description of EnDisease” with a statistical description of the collected DNase-seq experiments, and analyzed the number of disease-associated enhancers articles of each year in EnDisease.
3) We modified Section 2.4 “Analysis of disease-enhancers across chromatin accessibility annotations in different cell types” using the updated DNase-seq annotation. We re-analyzed and updated the results.
4) We updated Section 4.1.1 “Literature search and review” with a clear definition of our candidate disease vocabulary and showed a concrete searching example.
5) We added Section 4.1.2 “Inclusion criteria” to clarify the detailed criteria for enhancer-disease associations, enhancer-target gene associations and enhancer definitions.
6) We added Section 4.1.3 “Data annotations” to illustrate how we curated disease information, enhancer information, and publication information. Notably, we collected 649 human and 115 mouse DNase-seq open chromatin experiments, and run the standard DNase-seq processing pipeline from ENCODE to annotate the epigenomic states of enhancers.
7) We added Section 4.2.2 “External link construction” to provide detailed construction of three kinds of external links in EnDisease: 1) Pubmed website; 2) OMIM website; 3) UCSC website.

By addressing these comments, we feel that the quality of our paper has been significantly improved. The point-by-point responses to the reviewers’ comments are given below. The changes made to the manuscript are shown using the track changes mode in MS Word.

Thank you for your consideration of our work. We look forward to your reply.

Sincerely yours,

Wanwen Zeng, Xu Min and Rui Jiang

**Response to the comments of the reviewers:**

**Reviewer: 1**

Major issues

1) The authors’ statement regarding the novelty and the advantages of EnDisease vs. another enhancer-disease database (DiseaseEnhancer) are not satisfactory. Both databases perform manual curation of enhancer-disease associations. The authors state “However, DiseaseEnhancer did not further annotate enhancers with other epigenomic experiments and carry out downstream analysis. To bridge this gap, we develop EnDisease, …”
   a) The authors claim that they annotate enhancers with epigenomic data but this is not demonstrated in the database. Specifically, the authors should explain the following puzzle regarding the epigenomic annotation in EnDisease and the downstream analysis based on this data. This data, mentioned in the paper and used to generate figures 2 and 3, is not seen neither in the database web interface, nor in the download options (despite declaring this in section 2.2.3). The file that should provide this data on the download page includes a list of 871 DNase-seq experiments, without any relation to the entries in EnDisease. This is also in contradiction of further statements, e.g. “Thus, we develop EnDisease, … resource on diverse enhancer-disease associations across various species with comprehensive epigenomic annotation.”

**Answer:** Thank you for pointing out the question. To distinguish EnDisease from other databases, we further collected 649 DNase-seq open chromatin experiments for human and 115 DNase-seq experiments for mouse to annotate the epigenomic states of our disease enhancers. The number of experiments changed because we unified all our experiments and enhancers to the newest genome version hg38 and mm10. For each experiment, we first run the ENCODE processing pipeline as shown in **Figure R1** to obtain read counts in peak regions.

Then we followed literature (1) and proposed the following method to quantify the degree of chromatin accessibility (i.e., openness score) for an enhancer in an experiment, with the consideration that the score should be comparable across experiments. Briefly, given an enhancer of length *L*, we treated this enhancer as foreground and denoted the count of reads in the enhancer region by *X*. To remove the sequencing depth effect, we chose a background region with length $L_{0}$ centered at the enhancer and denoted the count of reads in this background window by *Y*. We chose $L_{0}$=1Mb as the length of background region (midpoint ± 5kb). The openness score is then formally defined as the fold change of read numbers per base pair and can be simply calculated as

$$o=\frac{(X+\delta)/L}{(Y+\delta)/L_{0}}$$

where δ is a pseudo-count (the default value of δ is 5 in our implementation).

For each experiment, we provided detailed information (e.g., ENCODE access ID, cell line etc.) in the “Download” page. For each enhancer, we further added a column named “annotation” in the “Browse” and “Search” pages. Once a user clicks the annotation link, he or she can download all annotation scores for the specific enhancer. Besides, we also provided the whole annotation matrix in the “Download” page with detailed row name and column name.

**Figure R1. ENCODE uniform processing pipeline of DNase-seq hypersensitive sites for paired-end reads.** We follow these steps below: 1) Preparation: bwa indexing and hotspot mappability/blacklist preparation (run once on hg38 or mm10 reference files); 2) Alignment by bwa; 3) Merging and Filtering of bams and QC analysis; 4) BAM evaluation script; 5) Peak/hotspot calling by hotspot.

b) The authors should pay attention to the fact that the other resource, DiseaseEnhancer, did map their curated records to external databases of annotated enhancers, based on epigenomics experiments, e.g. RoadMap.

**Answer:** Thank you for reminding us of the fact about DiseaseEnhancer. In the revision, we have rephrased our description of DiseaseEnhancer accordingly in the manuscript. We have first removed the description of DiseaseEnhancer in Introduction and then added a detailed point-by-point comparison between EnDisease and DiseaseEnhancer, regarding the annotation of enhancers, in Discussion.

c) In addition to said above, the authors should describe a comparison between EnDisease and DiseaseEnhancer, in terms of

Methodology/approach – how were entries curated, what were the inclusion criteria.

Results – comparison of the resulting entries

**Answer:** Thank you for reminding us of making a comparison between EnDisease and DiseaseEnhancer. In the revision, we have made a point-by-point comparison between EnDisease and DiseaseEnhancer based on your advice and added the detailed comparison in Discussion as follows.

i. **For methodology/approach:**

**DiseaseEnhancer:**

1) *Candidate publications*: The authors curated their entries using a list of keywords, such as ‘enhancer’, ‘regulatory element’, ‘risk loci’, ‘mutation’, ‘copy number’, ‘variant’, ‘predisposition’, ‘susceptibility’, ‘cancer’, ‘tumour’ and ‘disease’, and obtained publications from PubMed.

2) *Inclusion criteria*: The authors extracted information of the disease-associated enhancers that were verified strictly by a series of experiments, including validation of enhancers, phenotypic/mechanistic characterization of relevant genetic alterations, and determination of targets of enhancers.

3) *Database features*: DiseaseEnhancer includes basic enhancer information (i.e. genomic location and target genes); disease types; associated variants on the enhancer and their mediated phenotypes (i.e. gain/loss of enhancer and the alterations of transcription factor bindings).

**EnDisease:**

1) *Candidate publications*: We first searched Pubmed database using “enhancer”, “cancer”, “tumor”, “disease” and curated a part of publications. Then we curated a list of 8,759 OMIM diseases, and searched the specific disease name (e.g. ‘Crohn’s disease’) and ‘enhancer’ to obtain candidate publications.

2) *Inclusion criteria*: First, the authors of a candidate publication should perform downstream biological experiments to validate whether the variation of the enhancer indeed cause disease. There are different kinds of experiments: mouse experiments which are the most intuitive to see the phenotype directly, and other indirect experiments, such as reporter assays detecting the effects of enhancers on the status of pathogenic genes (aberrant regulation). EnDisease collected experimental validated enhancer-disease associations and experimental validated enhancer-pathogenesis gene-disease associations. Second, the authors of a candidate publication should provide the exact information of the enhancer, including the chromosome, start site, end site, reference genome.

3) *Database features*: EnDisease includes disease information (disease name, associated mutation information, such as GWAS risk SNPs), enhancer information (chromosome, strand, start site, end site, associated genes, species, reference genome, cell type), and publication information (PubMed ID) and annotation information (openness scores from 649 human DNase-seq experiments and 115 mouse DNase-seq experiments).

ii. **For results:**

1) *For enhancer-disease associations*: DiseaseEnhancer documents 1059 associations between 167 diseases and 1059 enhancers from 203 articles. In contrast, EnDisease documents 535 associations between 133 diseases and 454 enhancers from 199 articles. We defined the unique associations using the term “Pubmed ID:chromosome:start site-end site”. Totally, 202 associations are shared in EnDisease and DiseaseEnhancer. We found that the unique associations from DiseaseEnhancer mainly came from only a small number of publications. For example, 308 enhancer-disease associations come from a single paper (PMID=27723759). Similarly, 211 associations come from another paper (PMID=27869826), and 79 from a third paper (PMID=25910213). Furthermore, most of these enhancer-disease associations are predicted instead of experimental validated. For example, in the paper (PMID=27723759) (2), the authors proposed a chromatin structure–based method to predict recurrent noncoding mutations in cancer and only performed reporter assays for 10 mutations out of 308 predictions. Only 4 of these mutations caused a significant increase in luciferase activity. Still, DiseaseEnhancer considered all of these 308 enhancer-disease associations as experimental validated, shown in **Table R1** at the bottom of this response letter. The unique associations from EnDisease mainly came from other species (57 enhancer-disease associations are from other species) and unique publications.

2) *For annotation*: DiseaseEnhancer paid more attention to mutation information. The authors annotated the variant consequence and assessed the mutation frequency of disease-associated enhancers using mutation profiles from the International Cancer Genome Consortium (ICGC). As a contrast, EnDisease put its effort into epigenomic annotations. We collected 649 human DNase-seq open chromatin experiments and 115 mouse DNase-seq open chromatin experiments to annotate the epigenomic states of our disease enhancers.

In summary, DiseaseEnhancer and EnDisease used different ways to curate enhancer-disease associations. Besides, DiseaseEnhancer paid more attention to mutation information, while EnDisease put its effort into annotating epigenomic status of these disease enhancers.

2) Methods – data mining and processing
   a) A clear and consistent definition of the inclusion criteria is missing. “After confirming these experimentally validated enhancer-disease associations…”. Deciding which records raised by text mining should be further used as confirmed valid enhancer-disease records in the database is crucial. The authors should clearly state the inclusion criteria for records in the database, specifically with regards to which experimental validation in the evaluated publications were defined as valid for EnDisease, both for the enhancer definition, for the enhancer-target gene association, and for the enhancer-disease association. One remarkable example is PMID=23273978, for which the enhancer-disease association is not yet established in the mined publication.

**Answer:** Thank you for your advice and we are sorry for the unclear description of our methods. In the revision, we have updated the inclusion criteria in “Section 4.1.2 Inclusion criteria”.

*For enhancer-disease associations*, we confirmed them as experimental validated using two criteria. First, the authors should perform downstream biological experiments to validate whether the variation of the enhancer indeed causes disease rather than prediction. There are different kinds of experiments: mouse experiments which are the most intuitive to directly see the phenotype, and other indirect experiments, such as reporter assays detecting the effects of enhancers on the status of pathogenic genes (aberrant regulation). For example, DiseaseEnhancer curated 308 enhancer-disease associations from a single paper (PMID=27723759). However, the authors only performed reporter assays of 10 mutations and 4 of these mutations caused a significant increase in luciferase activity. Therefore, EnDisease only recorded these 4 enhancer-disease associations instead of 308. EnDisease collected experimental validated enhancer-disease associations and experimental validated enhancer-pathogenesis gene-disease associations. Second, the authors should provide the exact information of the enhancer, including the chromosome, start site, end site, reference genome.

*For enhancer-target gene associations*, the inclusion criteria are similar to the enhancer-disease associations. The authors should perform experiments to validate enhancer-target gene associations. For example, the authors should perform chromosome conformation capture based methods such as 3C, 4C, 5C and Hi-C, luciferase report assays and etc. If the authors did not perform detailed experiments, we would not record the enhancer-target gene associations.

*For enhancer definitions*, we strictly followed enhancer locations in the original publications. The authors should provide detailed information of enhancer including the chromosome, start site, end site, reference genome. If the authors did not provide these details in the main manuscript or supplementary material, we would discard the enhancer-disease information. As for the example (PMID=23273978), we considered that the p53, tumor suppressor gene, was related to tumorigenesis. The authors showed that deletion of a single enhancer locus could result in the aberrant regulation. For example, p53BER2, located at 9q33.1, is associated with loss of heterozygosity in human cancer (3,4), so we considered the enhancer was related to tumorigenesis.

b) Major concerns regarding the used disease vocabulary:
   i. It is not clear how the disease vocabulary was created based on OMIM. Which OMIM data was used (e.g. disease names, disease aliases, symptoms, etc’)?

**Answer:** We are sorry that our description of our disease vocabulary was not clear. In the revision, we have updated the description of our disease vocabulary in “Section 4.1.1 Literature search and review”.

Here, we further clarify the disease vocabulary to facilitate understanding. At first, we searched Pubmed using the keyword “enhancer” and “tumor/cancer/disease”. However, the resulted publications were too general and only a small number of them were describing the experimental validated enhancer-disease associations. Therefore, then we changed to more specific search using OMIM disease. The number of OMIM diseases was wrong in the last version of our manuscript. Actually, we searched a total of 8,759 OMIM disease terms. For each term, we searched using the OMIM disease titles, alternative titles and symbols, since there are many alternative ways to define one single disease. We do not use symptoms because many diseases might have similar symptoms.

For example, for the OMIM #103100 (**Figure R2)**, we searched [“ADIE PUPIL” and “enhancer”], [“ADIE SYNDROME” and “enhancer”], [“POORLY REACTING PUPILS” and “enhancer”] as the results of the term. Thank you for pointing out our mistake, and we updated the description of our disease vocabulary in our manuscript.

**Figure R2. Example of OMIM entry**. We searched Pubmed database using the OMIM disease titles, alternative titles, and symbols separately.

ii. It is not clear how searching pubmed for all OMIM diseases resulted with only 549 diseases. “Therefore, we only collected 549 diseases terms in the first step”

**Answer:** We are sorry for the typos. In the revision, we have modified the text as “Therefore, we only collected 8,759 diseases terms in the first step”.

iii. The authors should explain the following: 62 out of the 549 records in the database have a disease name not from OMIM (e.g. tumorigenesis). This contradicts the data mining description and the subsequent statement “Since our candidate diseases are restricted in OMIM database, we could access the corresponding web pages in OMIM through the OMIM IDs for each disease.”

**Answer:** As mentioned before, we first searched Pubmed using general keywords “enhancer” and “disease/cancer/tumour” and curated 87 enhancer-disease associations without using OMIM disease vocabulary. The rough searching only brought us a small number of curated enhancer-disease associations. Therefore, we then used the OMIM disease vocabulary and searched Pubmed using more precise keywords “OMIM disease title/alternative title/symbol” and “enhancer”. This helped us get the most of the associations. We are sorry that we missed the detailed information in our methods and we have updated the corresponding sections in this version.

Minor issues

1) Writing style and clarity. Text clarity, phrasing and grammar should be improved throughout the entire manuscript. Examples:
   a) In many places ‘chromatin’ is used instead of ‘chromosome’.
   b) This sentence should be rephrased: “Cancer-enhancers are permissive enhancers, they almost function in all cell types”
   c) “…enhancers are extremely important regulatory elements that lead to diseases…” Enhancers do not lead to diseases, genetic alterations of enhancer sequences might do so.

**Answer:** We feel apologetic about these typos in writing and we have revised the errors in this version.

a) Thank you for pointing out our misusing the word “chromatin”. We replaced “chromatin” with “chromosome” in Page 6, Page 7, Page 13, and Page 15.
b) We removed Section 2.4.1 “Cancer-enhancers are permissive enhancers, they almost function in all cell types” and performed analysis of disease-enhancers across chromatin accessibility annotations in different cell types.
c) We rephrased this sentence to “…enhancers are extremely important regulatory elements, and non-coding variants in these regions are likely to cause diseases”. We also rephrased the sentence in Page 4 line 10 from “…show that enhancers can cause diseases through a diverse range of molecular mechanisms.” to “…show that these variants in enhancers can cause diseases through a diverse range of molecular mechanisms”.
2) “Various Species”. The authors mention the support of various species, however in fact, for species other than human or mouse, only 20 records, originating from 4 curated publications and belonging to 9 organisms exist. It means that 9 organisms have between 1 and 3 records each. This should be clearly stated in text. This state of the database is in contradiction with statements claiming that EnDisease is a comprehensive database across various species, e.g. “To bridge this gap, we develop EnDisease, a manually curated database of experimentally supported enhancer and disease associations, which seeks to provide a high quality, comprehensive and specialist resource of enhancer-related deregulation across various species”.

**Answer:** Thank you for pointing this out. In the revision, we have removed words like “comprehensive” and “across various species” as you mentioned, in Page 5 line 19, 20 and 21, and in Discussion. We added the sentence “Besides for human and mouse studies, we collect 20 enhancer-disease associations in 9 species in case of the need for future cross-species studies.” in Page 5 line 21. Recently, an article (5) described the huge ‘cross-species gap’ between human and mouse and transfers the gene-level expression from mouse to human. The authors used 170 pairs of human-mouse data set to perform training from mouse expression profiles and prediction in human expression profiles. Despite the small numbers of other species in EnDisease, we expect some researchers can bridge enhancer-level ‘cross-species gap’ using our database.

3) Numerous instances of not clear results or their interpretation.
   a) The following chapter in the results section is not clear – “2.3 Overview for EnDisease”. Except the first paragraph, there are absolutely no results. In addition, the logic of how the conclusion of the following sentence is based on the results of this manuscript is not clear - “In summary, the statistical analysis for enhancer-disease associations in EnDisease database indicates that enhancers are extremely important regulatory elements that lead to diseases.”

**Answer:** Thank you for pointing out the unclear results. We rewrote Section 2.3 and modified the logical error and renamed Section 2.3 to “Statistical description of EnDisease”. We maintained the first paragraph unchanged claiming that most collected diseases were cancer-related. We substituted the second paragraph with statistical results of our collected DNase-seq experiments to show the comprehensive annotations of EnDisease. We updated the third paragraph with publication related statistics. In detail, we drew a histogram of the number of publications about enhancer-disease associations per year, to illustrate that more and more articles about enhancer-disease were published. We updated the conclusion in the last paragraph and demonstrated that more and more attention has been paid to enhancer-disease studies and EnDisease has collected and annotated these associations, which will be beneficial to further downstream study.

b) “We draw the distribution of the numbers of open cell types for all disease-enhancers in Figure 3 and find that more than half of the collected enhancers are cell type-specific”. Afterwards the authors claim that “…These results indicate that for most of the diseaseenhancers, they are very cell-type specific …”. The authors should explain how they quantitatively define cell type specific enhancers, and provide statistics, e.g. the number of cell type specific enhancers according to this definition, as opposed to stating ‘more than half’ or ‘most’ or ‘very’.

**Answer:** Thank you for pointing out the unclear description and we updated “Section 2.3 Statistical description of EnDisease” and “Section 2.4 Analysis of disease-enhancers across chromatin accessibility annotations in different cell types” with more detailed results. We considered an enhancer as open in a cell type, if this enhancer has an openness score larger than 2 in this cell type; we defined an enhancer as cell type-specific, if it is open in less than 37 (20% of collected cell lines) cell types.

In this version, we first introduced the definition of enhancer openness and enhancer cell type-specificity in the main manuscript. Then we updated the sentence to “We define an enhancer as open in a specific cell type if the enhancer has an openness score larger than 2 in the cell type; we define an enhancer as cell type-specific, if it is open in less than 37 (20% of collected cell lines) cell types; we define an enhancer as permissive, if it is open in more than 110 (60% of collected cell lines) cell types. We then draw the distribution of the numbers of open cell types for all disease-enhancers in Figure 3. from which we find that 53.51% enhancers are cell type-specific, while 5.32% enhancers are permissive”

c) “Especially, enhancers in cluster 6 are only open in a few cell types (less than 10 cell types in 202 cell types).”. It can be clearly seen that some rows in cluster 6 have more than 10 marked cell types. Please explain.
d) “We also extract the related disease and find that all the related diseases in cluster 6 are complex diseases, and the corresponding open cell types are related to the complex disease”. The authors should state how they compared the vocabulary of cells in the DNase data with the corresponding cells of the diseases, and show the entire comparison results to support the abovementioned claim “…the corresponding open cell types are related to the complex disease”.

**Answer:** We are sorry that we made the improper statement in the previous manuscript. According to the definition in answer (b), an enhancer is considered as open in a cell type, if it has an openness score larger than 2 in this cell type. Actually, the y-axis range of the bi-clustering figure is from 0 to 2, which makes the heatmap a little bit confusing.

In this version, we feel that a straightforward statistical test is more intuitive and informative to demonstrate the difference in cell type specificity between enhancers related and irrelevant to cancer. With this consideration, we have replaced the bi-clustering section with carefully designed statistical analysis as follows. First, we lift over all human enhancers in our database to the hg38 reference genome, and we re-run the ENCODE processing pipeline for all human DNase-seq experiments to obtain openness scores for these enhancers. Then, we define an enhancer as open in a specific cell type if the enhancer has an openness score larger than 2 in the cell type, define an enhancer as cell type-specific if the enhancer is open in less than 37 (20% of collected cell lines) cell types, and define an enhancer as permissive if the enhancer is open in more than 110 (60% of collected cell lines) cell types. We find that 53.51% enhancers are cell type-specific, while 5.32% enhancers are permissive. Furthermore, among these permissive enhancers, 77.27% (17 out of 22) of them are related to cancers. These results from simple statistical analysis suggest that cancer-related enhancers are likely to be permissive. To further validate this conjecture, we perform a one-sided Wilcoxon rank sum test against the alternative hypothesis that cancer-related enhancers are open in more cell types than enhancers irrelevant to cancer. The small *p*-value ($4.33\times{10}^{-9}$) demonstrated that cancer-related enhancers indeed function in more cell types than non-cancer related enhancers. We have updated our analysis and conclusion in Section 2.4. Hope the reviewer could agree with us that such statistical analysis is easier to understand and more convincing than the previous cluster analysis, and thus allow us to include this part in the main text (Section 2.4).

e) The authors should include the entire matrix, with values and row/column names for figure #2 in supplementary data.

**Answer:** We uploaded the entire annotation matrix in **Supplementary Table 4** with row name and column name annotated.

f) Figure 3 – the authors should explain how the number of open cell types was determined from the openness score.

**Answer:** As defined in answer (b), we considered an enhancer as open in a cell type, if it has an openness score larger than 2. We defined an enhancer as cell type-specific, if it is open in less than 37 (20% of collected cell lines) cell types.

g) Table 1 – The authors should explain how the number of enhancers in the ‘total’ line is not the sum of all species enhancers.

**Answer:** We really appreciate your advice. As you pointed out in the Minor issue 5 and 6, we lift over our genome version to the newest version and changed our database main key and recalculated the data statistic in **Table 1**. The number of enhancer-disease associations is the number of unique tuples of “Pubmed ID:OMIM ID:chromosome:start site-end site”. The number of enhancers is the number of unique tuples of “chromosome:start site-end site”. The number of diseases is the number of unique values of “OMIM ID”. The updated **Table 1** is shown below:

**Table 1. Statistics for enhancer-disease associations in the EnDisease database**. Each row represents a species. The enhancers column indicates the number of enhancers involved in this species, the diseases column indicates the number of diseases involved in this species and enhancer-disease associations columns indicates the number of enhancer-disease association in this species. The associations we collected are mainly from human. The last line represents the total number of enhancers, diseases, and enhancer-disease associations across species. Note that the total number of diseases is not a direct summation since some diseases are studied across different species.

| Species | enhancers | diseases | enhancer-disease associations |
| --- | --- | --- | --- |
| Homo sapiens | 413 | 125 | 483 |
| Mus musculus | 21 | 13 | 32 |
| Others  Total | 20  454 | 4  133 | 20  535 |

h) The authors should relate to the discrepancy with the number of publications in the database. In Section 2.1 the number is “203” while the abstract states “curated from more than 203 articles”

**Answer:** We removed “more than” in the abstract to keep consistency in our manuscript. After updating our enhancer-disease associations to newest genome version and removing some associations cannot be lifted over, the remaining enhancer-disease associations came from 199 of the articles. We will be more careful about such statements.

4) Methods are often lacking key details
   a) DNAse-seq. The authors should provide the source details and citation for this data. “We collected 871 DNase-seq experiments”.
   b) Methods – L0. The authors should clarify how the background region was selected and how L0 was calculated.

**Answer:** Thanks for reminding us of providing these key details.

a) We updated the citation for DNase-seq in Page 5 line 23 and further uploaded the detail information of each collected experiments in **Supplementary Table 3**. Since **Supplementary Table 3** has too many rows, we did not paste it here and just uploaded another separate Excel file in Supplementary material (649 human DNase-seq experiments and 115 mouse DNase-seq experiments).
b) For each enhancer region, we chose a background region with length $L_{0}$ centered at the enhancer. We set $L_{0}$=1Mb following the instructions in (1). We also updated the Methods section to clarify how the background region was selected and how $L_{0}$ was chosen.

5) Missing database features
   a) The authors should allow users to view the genomic coordinates of a given organism using a common genomic build.

**Answer:** Thank you for your advice. We have updated the genomic coordinates of all the enhancer-disease associations and updated the description of the external links construction in Supplementary Methods.

We lifted over human and mouse genome coordinates to the newest version (hg38 for Homo sapiens and mm10 for Mus musculus) and kept the genome coordinates for other species since liftover in these species might be inaccurate. There are 14 entries which cannot be lifted over successfully, so we removed them in this version. Besides, in the “Browse” and “Search” pages, we removed the column named “ref genome” and added **Supplementary Table 1** for reference genome for each species. In this way, when visualizing the enhancers in USCS genome browser, users will have consistent visualization by a common genomic coordinate. We attached **Supplementary Table 1** below:

**Supplementary Table 1. Reference genome for each species.** We lifted over human and mouse genome coordinates to the newest version (hg38 for Homo sapiens and mm10 for Mus musculus) and kept the genome coordinates for other species.

| Species | Reference genome |
| --- | --- |
| Homo sapiens | hg38 |
| Mus musculus | mm10 |
| Anolis carolinensis | anoCar1 |
| Bos taurus | bosTau4 |
| Drosophila melanogaster | dm6 |
| Gallus gallus | galGal3 |
| Ornithorhynchus anatinus | ornAna1 |
| Oryctolagus cuniculus | oryCun2 |
| Oryzias latipes | oryLat2 |
| Rattus norvegicus | rn6 |

b) The authors should describe ways to construct urls to link to this database entries/search results, for link-outs from external resources.

**Answer:** There are three kinds of external links in EnDisease: 1) Pubmed website; 2) OMIM website; 3) UCSC website. For Pubmed website, we extracted the Pubmed ID such as 6153459 for the entry and added it to the last of the link <https://www.ncbi.nlm.nih.gov/pubmed/> as <https://www.ncbi.nlm.nih.gov/pubmed/6153459/> and finished Pubmed link construction; For OMIM website, we extracted the OMIM ID such as 613985 for the entry and added it to the last of the link https://www.omim.org/entry/ as https://www.omim.org/entry/613985 and finished OMIM link construction; For UCSC visualization, we extracted the chromosome (chr11), start site (5155389), end site (5266609) and species (Homo sapiens) for the entry and mapped the species to reference genome (hg38) using **Supplementary Table 1.** We put this information to the link <http://genome.ucsc.edu/cgi-bin/hgTracks?db=(ref>erence genome)&position=(chromosome):(start site)-(end site) as <http://genome.ucsc.edu/cgi-bin/hgTracks?db=hg38&position=chr11:5155389-5266609> and finished UCSC link construction. We have added these external link construction methods in Supplementary Methods.

6) The authors define the major entry in the database (section 2.1) as an association between (1) enhancer; (2) disease; (3) publication.
   a) I suspect that the database statistics in section 2.3 are inaccurately presented. If an enhancer-disease association is reported by multiple publications, EnDisease creates a separate entry for each such association, hence the same enhancer-disease relation is counted multiple times if appears in multiple publications. One example is beta-Thalassaemia.
   b) This contradicts the methods statement “…table in MySQL to store all the information, with OMIM ID, chromosome, start site and end site as main key…”. In this statement the publication is not part of defining the main entry. The authors should be consistent with the definition of the records in the database. In addition, in either solution, it should be explained how cases where multiple publications report on identical enhancer-disease association are handled.

**Answer:** Thank you for pointing out the phenomenon in EnDisease. We are sorry for the mistakes in describing the main key in our database. First of all, we rephrased “…table in MySQL to store all the information, with OMIM ID, chromosome, start site and end site as main key…” to “…table in MySQL to store all the information, with Pubmed ID, OMIM ID, chromosome, start site and end site as primary key…”. At the current version of EnDisease, we changed the main key into the combination of 1) Pubmed ID 2) OMIM ID 3) chromosome 4) start site 5) end site as “Pubmed ID:OMIM ID:chromosome:start site-end site”.

Different articles might describe the same enhancer-disease associations studies in different ways (having different types of variations or affecting different genes), so we regarded them as different enhancer-disease associations. As you pointed out, the beta-Thalassaemia are recorded as “6153459:613985:chr11: 5207464-5214464” and “450109:613985:chr11:5207464-5214464”. Although these two articles validating the same enhancer-disease associations, the mechanisms of the enhancer are different. The former one is large deletion affecting HBB genes, while the latter one suppressed in cis of HBG1/ HBG2 expression. Another example is alpha-Thalassaemia. The alpha-Thalassaemia are recorded as “16728641:604131:chr16:102658-103864” and “19959666:604131:chr16:102658-103864”. However, in the former article, the variation affected HBA gene and caused alpha-Thalassaemia, while the variation in the latter article affected NME4 gene.

For the database statistics, we regarded the same enhancer-disease associations with different mechanisms as different enhancer-disease associations (all and only two examples are shown above). The number of enhancer-disease associations is the unique number of “Pubmed ID:OMIM ID:chromosome:start site-end site”. The number of enhancers is the unique number of “chromosome:start site-end site”. The number of diseases is the unique number of “OMIM ID”. Therefore, we updated the database statistics in section 2.3. We really appreciate your advice.

7) The authors should provide more details regarding the update plans for adding records to the database (e.g. update frequency).

**Answer:** Thank you for your advice and we also wish to update our database more frequently. However, two aspects limit update frequency. First, most of the enhancer-disease associations are dispersed in many independent studies. It might take several years to discover candidate enhancers set and validate some of them using different biological experiments such as mice experiment. Therefore, still a few articles (~50) will be published yearly. Second, the workflow of our database is 1) searching the Pubmed database for candidate articles; 2) overviewing the abstract for screening the ones validating enhancer-disease associations; 3) manually collecting the enhancer-disease associations from the articles and supplementary data; 4) checking with the authors if no detailed information is provided; 5) double checking our data. It also takes us a long time (up to several months) to perform the process. Therefore, we wish to update our database every two years.

**Reviewer: 2**

Major concerns

1) The enhancer coordinates are listed in their original. The authors should provide the data using the same genome version. I will strongly recommend using hg38 version for human genome data and mm10 for mouse genome data. And also use the latest version of genome annotations for rest of the species.
2) If I click the UCSC visualize icon for the annotations in the older version of genomes, then it takes me to hg38. It is because hg17 or mm5 are no longer supported on UCSC genome browser. The authors should liftover the genomic to the latest genome versions.

**Answer:** Thank you for your advice. In the revision, we have updated all the enhancer-disease associations using unified genome version.

We lifted over human and mouse genome coordinates to the newest version (hg38 for Homo sapiens and mm10 for Mus musculus) and kept the genome coordinates for other species since liftover in these species might be inaccurate. There are 14 entries which cannot be lifted over successfully, so we removed them in this version. Besides, in the “Browse” and “Search” pages, we removed the column named “ref genome” and add **Supplementary Table 1** to list reference genomes for all species. In this way, when visualizing the enhancers in USCS genome browser, users will have consistent visualization by a common genomic coordinate. We attached **Supplementary Table 1** below:

**Supplementary Table 1. Reference genome for each species.** We lifted over human and mouse genome coordinates to the newest version (hg38 for Homo sapiens and mm10 for Mus musculus) and kept the genome coordinates for other species.

| Species | Reference genome |
| --- | --- |
| Homo sapiens | hg38 |
| Mus musculus | mm10 |
| Anolis carolinensis | anoCar1 |
| Bos taurus | bosTau4 |
| Drosophila melanogaster | dm6 |
| Gallus gallus | galGal3 |
| Ornithorhynchus anatinus | ornAna1 |
| Oryctolagus cuniculus | oryCun2 |
| Oryzias latipes | oryLat2 |
| Rattus norvegicus | rn6 |

3) Given that there are different genome versions, I wonder how the authors performed the clustering analysis of these enhancers with the chromatin accessibility (DNase-seq data)?

**Answer:** We appreciate the reviewer for the pointing out this problem in our previous manuscript. Actually we lifted over human enhancers to hg19 and then performed annotation. In detail, we first run the DNase-seq processing pipeline (**Figure R1)** from ENCODE for all collected DNase-seq experiments to obtain enhancer openness scores. We then lifted over enhancers to hg19 to annotate the openness scores for each enhancer and performed bi-clustering.

In this version, we feel that a straightforward statistical test is more intuitive and informative to demonstrate the difference in cell type specificity between enhancers related and irrelevant to cancer. With this consideration, we have replaced the bi-clustering section with carefully designed statistical analysis as follows. First, we lift over all human enhancers in our database to the hg38 reference genome, and we re-run the ENCODE processing pipeline for all human DNase-seq experiments to obtain openness scores for these enhancers. Then, we define an enhancer as open in a specific cell type if the enhancer has an openness score larger than 2 in the cell type, define an enhancer as cell type-specific if the enhancer is open in less than 37 (20% of collected cell lines) cell types, and define an enhancer as permissive if the enhancer is open in more than 110 (60% of collected cell lines) cell types. We find that 53.51% enhancers are cell type-specific, while 5.32% enhancers are permissive. Furthermore, among these permissive enhancers, 77.27% (17 out of 22) of them are related to cancers. These results from simple statistical analysis suggest that cancer-related enhancers are likely to be permissive. To further validate this conjecture, we perform a one-sided Wilcoxon rank sum test against the alternative hypothesis that cancer-related enhancers are open in more cell types than enhancers irrelevant to cancer. The small *p*-value ($4.33\times{10}^{-9}$) demonstrated that cancer-related enhancers indeed function in more cell types than non-cancer related enhancers. We have updated our analysis and conclusion in Section 2.4. Hope the reviewer could agree with us that such statistical analysis is easier to understand and more convincing than the previous cluster analysis, and thus allow us to include this part in the main text (Section 2.4).

4) Through bi-clustering, the authors suggest that permissive enhancers are important in cancers while other cell-type specific enhancers are important in other diseases. It is known that enhancers work in cell-type specific fashion. Given this, does it mean the permissive enhancers are not cell-type specific?

**Answer:** We appreciate the reviewer for pointing out this problem in our previous manuscript. In the revision, we have updated Section 2.4, namely “Analysis of disease-enhancers across chromatin accessibility in different cell types,” to further discuss the cell-type specificity of enhancers. Enhancers, originally defined as cis-acting control elements, consist of ubiquitous, and in many cases cell type-specific, sequence cassettes (6). Most of the enhancers drive cell type-specific gene expression and some enhancers might be ubiquitous and make some genes, such as house-keeping genes, express constantly in different experimental conditions. Using STARR-seq, Zabidi *et al*. screened the whole fly genome with the use of different core promoters from either ubiquitously expressed housekeeping genes or developmentally regulated and cell-type-specific genes (7). They found that promoter-proximal enhancers are mainly ubiquitously expressed and regulate promoters of housekeeping genes, while promoters of developmental genes are cell type-specific and required distally located enhancers.

Minor concerns

1) There are many sub-sections in the results sections and also text such as, “we will discuss the results in the following section.”. Given this, the manuscript lakes a good flow of reading.

**Answer:** Thank you for the valuable suggestion. We have reorganized our manuscript in this version and made it easier to read.

2) This resource can be more useful after integrating additional data. Such as expression data for cancers from ICGC/TCGA, variant/SNP data at these enhancer regions etc.

**Answer:** Thank you for your advice. To distinguish EnDisease from other databases, we further collected 649 human and 115 mouse DNase-seq open chromatin experiments to annotate the epigenomic states of these enhancers. We provided these experimental annotations in the Download section. Besides, we also provided external links to ICGC/TCGA website to for readers to get more information.

3) Table 2 should be moved to supplementary.

**Answer:** In the revision, we have moved Table 2 into the supplementary file as **Supplementary Table 2**.

4) There is a typo in Figure 3 labels “Then number..”. Also the legends are not detailed.
5) The manuscript needs good attention. I recommend the authors to ask some native English speaker to go over the manuscript.

**Answer:** We feel apologetic about these typos in writing and we have further checked the manuscript and fixed mistakes in this version.

**Reviewer: 3**

Major revision:

1) Page 5: The authors said that DiseaseEnhancer did not annotate enhancers with other epigenomic experiments, while this endisease database provides the experiment data. It might be one of main advantages of endisease compared to DiseaseEnhancer. In endisease, epigenomic experiments are provided in the download file (http://bioinfo.au.tsinghua.edu.cn/endisease/download_dataset.php). However, this file does not provide which enhancer-disease associations are supported by these experiments. In addition to the downloadable file, it might be better if this information can be browsed with disease-enhancer information.

**Answer:** Thank you for pointing out the question. In the revision, we have updated the manuscript and updated the “Browse”, “Search” and “Download” pages of EnDisease website. To distinguish EnDisease from others, we further collected 649 human and 115 mouse DNase-seq open chromatin experiments to annotate the epigenomic states of these enhancers. For each experiment, such as DNase-seq, we first run the ENCODE processing pipeline (**Figure R1**). We then annotated the openness scores for each enhancer. We provided detailed information such as ENCODE access ID and cell line, in the “Download” page. For each enhancer, we further added one column named “annotation” in the “Browse” and “Search” pages. Once a user clicks the annotation link, he or she can download all annotation score for the specific enhancer. Besides, we also provided the whole annotation matrix in the “Download” page with detailed row name and column name.

2) How many unique enhancer-disease associations are provided compared to DiseaseEnhancer? It might be good if statistical comparisons among databases with enhancer-disease associations are provided.

**Answer:** In the revision, we have followed the suggestion and compared our database with DiseaseEnhancer in a comprehensive way, as follows.

*For enhancer-disease associations*: DiseaseEnhancer documents 1059 associations between 167 diseases and 1059 enhancers curated from 203 articles. In contrast, EnDisease documents 535 associations between 133 diseases and 454 enhancers curated from 199 articles. We defined the unique associations using the term “Pubmed ID:chromosome:start site-end site”. Totally, 202 associations are shared in EnDisease and DiseaseEnhancer. We found that the unique associations from DiseaseEnhancer mainly came from only a few publications. For example, 308 enhancer-disease associations are from a single paper (PMID=27723759), 211 from PMID=27869826, 79 associations from PMID=25910213. Most of these enhancer-disease associations are predicted, not experimental validated. For example, in PMID=27723759 (2), the authors proposed a chromatin structure–based methods to predict 308 recurrent noncoding mutations in cancer and only performed reporter assays of 10 mutations. Only 4 of these mutations caused a significant increase in luciferase activity. However, DiseaseEnhancer considered all of these as experimental validated enhancer-disease associations as shown in Table R1. The unique associations from EnDisease mainly came from other species (57 enhancer-disease associations are from other species) and unique publications.

*For annotation*: DiseaseEnhancer paid more attention to mutation information. The authors annotated the variant consequence and assessed the mutation frequency of disease-associated enhancers using mutation profiles from International Cancer Genome Consortium (ICGC). In contrast, EnDisease put more effort into epigenomic annotations. We collected 649 human DNase-seq open chromatin experiments and 115 mouse DNase-seq open chromatin experiments to annotate the epigenomic states of our disease enhancers.

3) Page 8: In Section 2.3, the authors mentioned that “the number of disease-associated enhancers articles keeps increasing year by year,” It would be good if these paragraph is supported with statistics.

**Answer:** Thank you for your advice. In the revision, we have drawn the distribution of the number of publications per year. By analyzing the entries from EnDisease database, we can find the publications about the aberrantly expressed enhancers related with diseases are increasing yearly (**Figure R3**), which indicates the investigation of the associations between enhancers and diseases is becoming one of the hottest topics.

**Figure R3. The number of entries in recent years.** The number of disease-associated enhancers articles keeps increasing year by year.

Minors:

1) Page 6 line 30: strand is repeated twice.
2) Figure 2: Provide labels for x-axis and y-axis.
3) Figure 3: in x-axis, “Then number of ...” -> “The number of …”
4) Figure 3: Provide more detailed label for y-axis.

Answer: We feel apologetic about these typos in writing and we have revised them in this version.

**Reference**

1.	Duren, Z., Chen, X., Jiang, R., Wang, Y. and Wong, W.H. (2017) Modeling gene regulation from paired expression and chromatin accessibility data. *Proc Natl Acad Sci U S A*, **114**, E4914-E4923.

2.	Kim, K., Jang, K., Yang, W., Choi, E.Y., Park, S.M., Bae, M., Kim, Y.J. and Choi, J.K. (2016) Chromatin structure-based prediction of recurrent noncoding mutations in cancer. *Nat Genet*, **48**, 1321-1326.

3.	Callahan, G., Denison, S.R., Phillips, L.A., Shridhar, V. and Smith, D.I. (2003) Characterization of the common fragile site FRA9E and its potential role in ovarian cancer. *Oncogene*, **22**, 590-601.

4.	Schultz, D.C., Vanderveer, L., Buetow, K.H., Boente, M.P., Ozols, R.F., Hamilton, T.C. and Godwin, A.K. (1995) Characterization of chromosome 9 in human ovarian neoplasia identifies frequent genetic imbalance on 9q and rare alterations involving 9p, including CDKN2. *Cancer Res*, **55**, 2150-2157.

5.	Normand, R., Du, W., Briller, M., Gaujoux, R., Starosvetsky, E., Ziv-Kenet, A., Shalev-Malul, G., Tibshirani, R.J. and Shen-Orr, S.S. (2018) Found In Translation: a machine learning model for mouse-to-human inference. *Nat Methods*, **15**, 1067-1073.

6.	Voss, B., Glossl, J., Cully, Z. and Kresse, H. (1986) Immunocytochemical investigation on the distribution of small chondroitin sulfate-dermatan sulfate proteoglycan in the human. *J Histochem Cytochem*, **34**, 1013-1019.

7.	Zabidi, M.A., Arnold, C.D., Schernhuber, K., Pagani, M., Rath, M., Frank, O. and Stark, A. (2015) Enhancer-core-promoter specificity separates developmental and housekeeping gene regulation. *Nature*, **518**, 556-559.

**Appendix**

**Table R1. 308 enhancer-disease associations in the paper (PMID=27723759)** **from DiseaseEnhancer 1.0.2.**

| ID | chr | start | end | gene | PMID |
| --- | --- | --- | --- | --- | --- |
| DE_00042 | chr10 | 6244293 | 6244293 | GATA3 | 27723759 |
| DE_00044 | chr10 | 7567024 | 7567024 | GATA3 | 27723759 |
| DE_00045 | chr10 | 7928151 | 7928151 | GATA3 | 27723759 |
| DE_00046 | chr10 | 8067866 | 8067866 | GATA3 | 27723759 |
| DE_00047 | chr10 | 8102520 | 8102520 | GATA3 | 27723759 |
| DE_00048 | chr10 | 8374958 | 8374958 | GATA3 | 27723759 |
| DE_00049 | chr10 | 8393839 | 8393839 | GATA3 | 27723759 |
| DE_00050 | chr10 | 8402402 | 8402402 | GATA3 | 27723759 |
| DE_00051 | chr10 | 8405624 | 8405624 | GATA3 | 27723759 |
| DE_00052 | chr10 | 8899177 | 8899177 | GATA3 | 27723759 |
| DE_00053 | chr10 | 8899312 | 8899312 | GATA3 | 27723759 |
| DE_00054 | chr10 | 8900652 | 8900652 | GATA3 | 27723759 |
| DE_00055 | chr10 | 8903379 | 8903379 | GATA3 | 27723759 |
| DE_00056 | chr10 | 8911062 | 8911062 | GATA3 | 27723759 |
| DE_00060 | chr10 | 89872831 | 89872831 | PTEN | 27723759 |
| DE_00061 | chr10 | 89913137 | 89913137 | PTEN | 27723759 |
| DE_00062 | chr10 | 89938170 | 89938170 | PTEN | 27723759 |
| DE_00063 | chr10 | 90146219 | 90146219 | PTEN | 27723759 |
| DE_00064 | chr10 | 9079498 | 9079498 | GATA3 | 27723759 |
| DE_00065 | chr10 | 9112313 | 9112313 | GATA3 | 27723759 |
| DE_00066 | chr10 | 9117103 | 9117103 | GATA3 | 27723759 |
| DE_00067 | chr10 | 9201133 | 9201133 | GATA3 | 27723759 |
| DE_00068 | chr10 | 9215998 | 9215998 | GATA3 | 27723759 |
| DE_00069 | chr10 | 9343954 | 9343954 | GATA3 | 27723759 |
| DE_00071 | chr11 | 107880536 | 107880536 | ATM | 27723759 |
| DE_00072 | chr11 | 108211845 | 108211845 | ATM | 27723759 |
| DE_00073 | chr11 | 109962688 | 109962688 | ATM | 27723759 |
| DE_00078 | chr1 | 115300152 | 115300152 | NRAS | 27723759 |
| DE_00079 | chr1 | 115300224 | 115300224 | NRAS | 27723759 |
| DE_00092 | chr11 | 68859692 | 68859692 | CCND1 | 27723759 |
| DE_00093 | chr11 | 68872778 | 68872778 | CCND1 | 27723759 |
| DE_00094 | chr11 | 68881643 | 68881643 | CCND1 | 27723759 |
| DE_00095 | chr11 | 68886052 | 68886052 | CCND1 | 27723759 |
| DE_00096 | chr11 | 69051978 | 69051978 | CCND1 | 27723759 |
| DE_00097 | chr11 | 69053154 | 69053154 | CCND1 | 27723759 |
| DE_00098 | chr11 | 69088100 | 69088100 | CCND1 | 27723759 |
| DE_00099 | chr11 | 69191851 | 69191851 | CCND1 | 27723759 |
| DE_00100 | chr11 | 69243913 | 69243913 | CCND1 | 27723759 |
| DE_00101 | chr11 | 69252451 | 69252451 | CCND1 | 27723759 |
| DE_00102 | chr11 | 69256005 | 69256005 | CCND1 | 27723759 |
| DE_00103 | chr11 | 69256008 | 69256008 | CCND1 | 27723759 |
| DE_00104 | chr11 | 69256011 | 69256011 | CCND1 | 27723759 |
| DE_00105 | chr11 | 69258373 | 69258373 | CCND1 | 27723759 |
| DE_00106 | chr11 | 69279835 | 69279835 | CCND1 | 27723759 |
| DE_00107 | chr11 | 69282363 | 69282363 | CCND1 | 27723759 |
| DE_00108 | chr11 | 69287640 | 69287640 | CCND1 | 27723759 |
| DE_00109 | chr11 | 69305144 | 69305144 | CCND1 | 27723759 |
| DE_00110 | chr11 | 69305799 | 69305799 | CCND1 | 27723759 |
| DE_00111 | chr11 | 69312746 | 69312746 | CCND1 | 27723759 |
| DE_00112 | chr11 | 69315935 | 69315935 | CCND1 | 27723759 |
| DE_00113 | chr11 | 69317977 | 69317977 | CCND1 | 27723759 |
| DE_00114 | chr11 | 69318175 | 69318175 | CCND1 | 27723759 |
| DE_00115 | chr11 | 69324989 | 69324989 | CCND1 | 27723759 |
| DE_00116 | chr11 | 69330742 | 69330742 | CCND1 | 27723759 |
| DE_00117 | chr11 | 69332416 | 69332416 | CCND1 | 27723759 |
| DE_00118 | chr11 | 69345852 | 69345852 | CCND1 | 27723759 |
| DE_00119 | chr11 | 69351959 | 69351959 | CCND1 | 27723759 |
| DE_00120 | chr11 | 69355586 | 69355586 | CCND1 | 27723759 |
| DE_00121 | chr11 | 69390451 | 69390451 | CCND1 | 27723759 |
| DE_00122 | chr11 | 69416853 | 69416853 | CCND1 | 27723759 |
| DE_00123 | chr11 | 69418111 | 69418111 | CCND1 | 27723759 |
| DE_00124 | chr11 | 69431690 | 69431690 | CCND1 | 27723759 |
| DE_00125 | chr11 | 69465198 | 69465198 | CCND1 | 27723759 |
| DE_00136 | chr12 | 115112467 | 115112467 | TBX3 | 27723759 |
| DE_00137 | chr12 | 11883580 | 11883580 | ETV6 | 27723759 |
| DE_00138 | chr12 | 12038275 | 12038275 | ETV6 | 27723759 |
| DE_00141 | chr12 | 12876142 | 12876142 | CDKN1B | 27723759 |
| DE_00142 | chr12 | 12879708 | 12879708 | ETV6 | 27723759 |
| DE_00143 | chr12 | 12957277 | 12957277 | CDKN1B | 27723759 |
| DE_00144 | chr12 | 50451445 | 50451445 | SMARCD1 | 27723759 |
| DE_00145 | chr1 | 26438008 | 26438008 | ARID1A | 27723759 |
| DE_00147 | chr1 | 26798607 | 26798607 | ARID1A | 27723759 |
| DE_00148 | chr1 | 27098601 | 27098601 | ARID1A | 27723759 |
| DE_00149 | chr1 | 27492915 | 27492915 | ARID1A | 27723759 |
| DE_00150 | chr1 | 27720081 | 27720081 | ARID1A | 27723759 |
| DE_00152 | chr13 | 32895005 | 32895005 | BRCA2 | 27723759 |
| DE_00153 | chr13 | 33213838 | 33213838 | BRCA2 | 27723759 |
| DE_00154 | chr13 | 47128005 | 47128005 | RB1 | 27723759 |
| DE_00156 | chr13 | 48611842 | 48611842 | RB1 | 27723759 |
| DE_00157 | chr13 | 48668171 | 48668171 | RB1 | 27723759 |
| DE_00158 | chr13 | 48669381 | 48669381 | RB1 | 27723759 |
| DE_00159 | chr13 | 49067569 | 49067569 | RB1 | 27723759 |
| DE_00160 | chr13 | 50656563 | 50656563 | RB1 | 27723759 |
| DE_00161 | chr14 | 105217554 | 105217554 | AKT1 | 27723759 |
| DE_00162 | chr14 | 105222648 | 105222648 | AKT1 | 27723759 |
| DE_00163 | chr14 | 105269669 | 105269669 | AKT1 | 27723759 |
| DE_00164 | chr14 | 105444532 | 105444532 | AKT1 | 27723759 |
| DE_00165 | chr14 | 37710185 | 37710185 | FOXA1 | 27723759 |
| DE_00166 | chr14 | 37710806 | 37710806 | FOXA1 | 27723759 |
| DE_00167 | chr14 | 37723745 | 37723745 | FOXA1 | 27723759 |
| DE_00168 | chr14 | 37733220 | 37733220 | FOXA1 | 27723759 |
| DE_00169 | chr14 | 38058268 | 38058268 | FOXA1 | 27723759 |
| DE_00170 | chr14 | 38118241 | 38118241 | FOXA1 | 27723759 |
| DE_00171 | chr14 | 38120099 | 38120099 | FOXA1 | 27723759 |
| DE_00172 | chr14 | 38126241 | 38126241 | FOXA1 | 27723759 |
| DE_00173 | chr14 | 38164005 | 38164005 | FOXA1 | 27723759 |
| DE_00174 | chr14 | 38167448 | 38167448 | FOXA1 | 27723759 |
| DE_00175 | chr14 | 38179158 | 38179158 | FOXA1 | 27723759 |
| DE_00176 | chr14 | 38182676 | 38182676 | FOXA1 | 27723759 |
| DE_00177 | chr14 | 38182684 | 38182684 | FOXA1 | 27723759 |
| DE_00178 | chr14 | 38182915 | 38182915 | FOXA1 | 27723759 |
| DE_00179 | chr14 | 38184293 | 38184293 | FOXA1 | 27723759 |
| DE_00189 | chr16 | 68738472 | 68738472 | CDH1 | 27723759 |
| DE_00190 | chr16 | 68741288 | 68741288 | CDH1 | 27723759 |
| DE_00191 | chr16 | 68750191 | 68750191 | CDH1 | 27723759 |
| DE_00192 | chr16 | 68752380 | 68752380 | CDH1 | 27723759 |
| DE_00193 | chr16 | 68823620 | 68823620 | CDH1 | 27723759 |
| DE_00194 | chr17 | 16126784 | 16126784 | NCOR1 | 27723759 |
| DE_00195 | chr17 | 16128122 | 16128122 | NCOR1 | 27723759 |
| DE_00200 | chr17 | 41445837 | 41445837 | BRCA1 | 27723759 |
| DE_00201 | chr17 | 56012996 | 56012996 | RAD51C | 27723759 |
| DE_00202 | chr17 | 56405056 | 56405056 | RAD51C | 27723759 |
| DE_00203 | chr17 | 56491423 | 56491423 | RAD51C | 27723759 |
| DE_00204 | chr17 | 56565448 | 56565448 | RAD51C | 27723759 |
| DE_00205 | chr17 | 56686893 | 56686893 | RAD51C | 27723759 |
| DE_00206 | chr17 | 56687036 | 56687036 | RAD51C | 27723759 |
| DE_00207 | chr17 | 56727563 | 56727563 | RAD51C | 27723759 |
| DE_00208 | chr17 | 56819395 | 56819395 | RAD51C | 27723759 |
| DE_00209 | chr17 | 56841079 | 56841079 | RAD51C | 27723759 |
| DE_00210 | chr17 | 57030283 | 57030283 | RAD51C | 27723759 |
| DE_00211 | chr17 | 57232982 | 57232982 | RAD51C | 27723759 |
| DE_00212 | chr17 | 57287378 | 57287378 | RAD51C | 27723759 |
| DE_00213 | chr17 | 57288400 | 57288400 | RAD51C | 27723759 |
| DE_00214 | chr17 | 57923238 | 57923238 | RAD51C | 27723759 |
| DE_00215 | chr17 | 59366414 | 59366414 | BRIP1 | 27723759 |
| DE_00216 | chr17 | 59376306 | 59376306 | BRIP1 | 27723759 |
| DE_00217 | chr17 | 59377191 | 59377191 | BRIP1 | 27723759 |
| DE_00218 | chr17 | 59525138 | 59525138 | BRIP1 | 27723759 |
| DE_00219 | chr17 | 59715852 | 59715852 | BRIP1 | 27723759 |
| DE_00220 | chr17 | 59722479 | 59722479 | BRIP1 | 27723759 |
| DE_00221 | chr17 | 59722480 | 59722480 | BRIP1 | 27723759 |
| DE_00222 | chr17 | 59789086 | 59789086 | BRIP1 | 27723759 |
| DE_00228 | chr17 | 6938079 | 6938079 | TP53 | 27723759 |
| DE_00229 | chr17 | 6939213 | 6939213 | TP53 | 27723759 |
| DE_00231 | chr17 | 7465409 | 7465409 | TP53 | 27723759 |
| DE_00232 | chr17 | 7534110 | 7534110 | TP53 | 27723759 |
| DE_00233 | chr17 | 7577498 | 7577498 | TP53 | 27723759 |
| DE_00234 | chr17 | 7621215 | 7621215 | TP53 | 27723759 |
| DE_00235 | chr17 | 8023124 | 8023124 | TP53 | 27723759 |
| DE_00236 | chr17 | 8026793 | 8026793 | TP53 | 27723759 |
| DE_00247 | chr20 | 55684882 | 55684882 | BRIP1 | 27723759 |
| DE_00248 | chr20 | 55701207 | 55701207 | BRIP1 | 27723759 |
| DE_00249 | chr20 | 55702731 | 55702731 | BRIP1 | 27723759 |
| DE_00250 | chr20 | 55774343 | 55774343 | BRIP1 | 27723759 |
| DE_00251 | chr20 | 55795637 | 55795637 | BRIP1 | 27723759 |
| DE_00252 | chr20 | 55829393 | 55829393 | BRIP1 | 27723759 |
| DE_00254 | chr2 | 202313604 | 202313604 | CASP8 | 27723759 |
| DE_00255 | chr2 | 202900229 | 202900229 | CASP8 | 27723759 |
| DE_00260 | chr22 | 29196386 | 29196386 | CHEK2 | 27723759 |
| DE_00261 | chr22 | 29196404 | 29196404 | CHEK2 | 27723759 |
| DE_00263 | chr22 | 29205398 | 29205398 | CHEK2 | 27723759 |
| DE_00286 | chr3 | 179169002 | 179169002 | PIK3CA | 27723759 |
| DE_00287 | chr3 | 179169300 | 179169300 | PIK3CA | 27723759 |
| DE_00288 | chr3 | 179169404 | 179169404 | PIK3CA | 27723759 |
| DE_00289 | chr3 | 184919694 | 184919694 | MAP3K13 | 27723759 |
| DE_00290 | chr3 | 185216716 | 185216716 | MAP3K13 | 27723759 |
| DE_00308 | chr3 | 52211851 | 52211851 | PBRM1 | 27723759 |
| DE_00309 | chr3 | 52274776 | 52274776 | PBRM1 | 27723759 |
| DE_00310 | chr3 | 52726385 | 52726385 | PBRM1 | 27723759 |
| DE_00316 | chr5 | 131826021 | 131826021 | RAD50 | 27723759 |
| DE_00317 | chr5 | 131838005 | 131838005 | RAD50 | 27723759 |
| DE_00318 | chr5 | 131892812 | 131892812 | RAD50 | 27723759 |
| DE_00319 | chr5 | 131898457 | 131898457 | RAD50 | 27723759 |
| DE_00321 | chr5 | 132109775 | 132109775 | RAD50 | 27723759 |
| DE_00328 | chr5 | 55711489 | 55711489 | MAP3K1 | 27723759 |
| DE_00329 | chr5 | 55724929 | 55724929 | MAP3K1 | 27723759 |
| DE_00330 | chr5 | 55786238 | 55786238 | MAP3K1 | 27723759 |
| DE_00331 | chr5 | 55832915 | 55832915 | MAP3K1 | 27723759 |
| DE_00332 | chr5 | 55969689 | 55969689 | MAP3K1 | 27723759 |
| DE_00333 | chr6 | 150465639 | 150465639 | ESR1 | 27723759 |
| DE_00335 | chr6 | 151935148 | 151935148 | ESR1 | 27723759 |
| DE_00336 | chr6 | 151937492 | 151937492 | ESR1 | 27723759 |
| DE_00338 | chr6 | 151957875 | 151957875 | ESR1 | 27723759 |
| DE_00339 | chr6 | 151958185 | 151958185 | ESR1 | 27723759 |
| DE_00340 | chr6 | 151961699 | 151961699 | ESR1 | 27723759 |
| DE_00342 | chr6 | 152232725 | 152232725 | ESR1 | 27723759 |
| DE_00344 | chr6 | 156984421 | 156984421 | ARID1B | 27723759 |
| DE_00345 | chr6 | 157041328 | 157041328 | ARID1B | 27723759 |
| DE_00346 | chr6 | 157110896 | 157110896 | ARID1B | 27723759 |
| DE_00347 | chr6 | 157154547 | 157154547 | ARID1B | 27723759 |
| DE_00348 | chr6 | 157389974 | 157389974 | ARID1B | 27723759 |
| DE_00359 | chr7 | 115956171 | 115956171 | MET | 27723759 |
| DE_00360 | chr7 | 115994876 | 115994876 | MET | 27723759 |
| DE_00361 | chr7 | 116312444 | 116312444 | MET | 27723759 |
| DE_00362 | chr7 | 116312573 | 116312573 | MET | 27723759 |
| DE_00363 | chr7 | 116314193 | 116314193 | MET | 27723759 |
| DE_00364 | chr7 | 116911021 | 116911021 | MET | 27723759 |
| DE_00365 | chr7 | 139695303 | 139695303 | BRAF | 27723759 |
| DE_00366 | chr7 | 140845142 | 140845142 | BRAF | 27723759 |
| DE_00370 | chr7 | 55102219 | 55102219 | EGFR | 27723759 |
| DE_00371 | chr7 | 55322571 | 55322571 | EGFR | 27723759 |
| DE_00373 | chr8 | 117778780 | 117778780 | RAD21 | 27723759 |
| DE_00374 | chr8 | 118742743 | 118742743 | RAD21 | 27723759 |
| DE_00385 | chr8 | 91585087 | 91585087 | NBN | 27723759 |
| DE_00387 | chr9 | 139414453 | 139414453 | NOTCH1 | 27723759 |
| DE_00388 | chr9 | 139460020 | 139460020 | NOTCH1 | 27723759 |
| DE_00389 | chr9 | 139492936 | 139492936 | NOTCH1 | 27723759 |
| DE_00390 | chr9 | 139512789 | 139512789 | NOTCH1 | 27723759 |
| DE_00391 | chr9 | 139929812 | 139929812 | NOTCH1 | 27723759 |
| DE_00672 | chr7 | 982031 | 982031 | ADAP1 | 27723759 |
| DE_00673 | chr20 | 46602388 | 46602388 | SULF2 | 27723759 |
| DE_00674 | chr20 | 46618545 | 46618545 | SULF2 | 27723759 |
| DE_00675 | chr20 | 52748710 | 52748710 | SULF2 | 27723759 |
| DE_00676 | chr20 | 46602388 | 46602388 | SULF2 | 27723759 |
| DE_00677 | chr20 | 46472698 | 46472698 | SULF2 | 27723759 |
| DE_00678 | chr20 | 46308627 | 46308627 | SULF2 | 27723759 |
| DE_00679 | chr20 | 46831426 | 46831426 | SULF2 | 27723759 |
| DE_00680 | chr20 | 46779797 | 46779797 | SULF2 | 27723759 |
| DE_00681 | chr20 | 46800093 | 46800093 | SULF2 | 27723759 |
| DE_00682 | chr20 | 47364914 | 47364914 | SULF2 | 27723759 |
| DE_00683 | chr20 | 46688376 | 46688376 | SULF2 | 27723759 |
| DE_00684 | chr20 | 45989659 | 45989659 | SULF2 | 27723759 |
| DE_00685 | chr4 | 49126582 | 49126582 | OCIAD2 | 27723759 |
| DE_00686 | chr4 | 49141001 | 49141001 | OCIAD2 | 27723759 |
| DE_00687 | chr4 | 49142162 | 49142162 | OCIAD2 | 27723759 |
| DE_00688 | chr20 | 62085791 | 62085791 | NKAIN4 | 27723759 |
| DE_00689 | chr20 | 62133292 | 62133292 | NKAIN4 | 27723759 |
| DE_00690 | chr20 | 61962472 | 61962472 | NKAIN4 | 27723759 |
| DE_00691 | chr20 | 61885983 | 61885983 | NKAIN4 | 27723759 |
| DE_00692 | chr20 | 61806945 | 61806945 | NKAIN4 | 27723759 |
| DE_00693 | chr10 | 375539 | 375539 | ZMYND11 | 27723759 |
| DE_00694 | chr10 | 127618 | 127618 | ZMYND11 | 27723759 |
| DE_00695 | chr12 | 53614168 | 53614168 | RARG | 27723759 |
| DE_00696 | chr12 | 53689776 | 53689776 | RARG | 27723759 |
| DE_00697 | chr12 | 53765398 | 53765398 | RARG | 27723759 |
| DE_00698 | chr20 | 46182305 | 46182305 | SULF2 | 27723759 |
| DE_00699 | chr1 | 2615274 | 2615274 | PANK4 | 27723759 |
| DE_00700 | chr1 | 2617111 | 2617111 | PANK4 | 27723759 |
| DE_00701 | chr1 | 2246358 | 2246358 | PANK4 | 27723759 |
| DE_00702 | chr16 | 30669784 | 30669784 | C16orf93 | 27723759 |
| DE_00703 | chr16 | 30669784 | 30669784 | C16orf93, SRCAP, PHKG2 | 27723759 |
| DE_00704 | chr16 | 30753142 | 30753142 | C16orf93 | 27723759 |
| DE_00705 | chr16 | 30753142 | 30753142 | C16orf93, SRCAP, PHKG2 | 27723759 |
| DE_00706 | chr16 | 30935409 | 30935409 | C16orf93, PHKG2 | 27723759 |
| DE_00707 | chr16 | 31153547 | 31153547 | C16orf93 | 27723759 |
| DE_00708 | chr16 | 31153547 | 31153547 | C16orf93 | 27723759 |
| DE_00709 | chr16 | 31153547 | 31153547 | C16orf93, SRCAP, PHKG2 | 27723759 |
| DE_00710 | chr17 | 81153381 | 81153381 | METRNL | 27723759 |
| DE_00711 | chr17 | 81153381 | 81153381 | METRNL | 27723759 |
| DE_00712 | chr20 | 52703188 | 52703188 | SULF2 | 27723759 |
| DE_00713 | chr20 | 52703188 | 52703188 | SULF2 | 27723759 |
| DE_00714 | chr16 | 29911922 | 29911922 | ZNF48 | 27723759 |
| DE_00715 | chr16 | 30346156 | 30346156 | ZNF48 | 27723759 |
| DE_00716 | chr16 | 30346156 | 30346156 | ZNF48, SRCAP | 27723759 |
| DE_00717 | chr16 | 30723097 | 30723097 | SRCAP | 27723759 |
| DE_00718 | chr16 | 30747922 | 30747922 | SRCAP | 27723759 |
| DE_00719 | chr8 | 145913001 | 145913001 | ZNF517, RPL8 | 27723759 |
| DE_00720 | chr4 | 190959438 | 190959438 | FRG2 | 27723759 |
| DE_00721 | chr1 | 2475021 | 2475021 | PEX10 | 27723759 |
| DE_00722 | chr1 | 2475021 | 2475021 | PEX10 | 27723759 |
| DE_00723 | chr1 | 2348267 | 2348267 | PEX10 | 27723759 |
| DE_00724 | chr1 | 2246358 | 2246358 | PEX10 | 27723759 |
| DE_00725 | chr20 | 60909815 | 60909815 | LAMA5 | 27723759 |
| DE_00726 | chr20 | 60999979 | 60999979 | LAMA5 | 27723759 |
| DE_00727 | chr20 | 61686432 | 61686432 | LAMA5 | 27723759 |
| DE_00728 | chr20 | 60878221 | 60878221 | LAMA5 | 27723759 |
| DE_00729 | chr12 | 52345023 | 52345023 | KRT80 | 27723759 |
| DE_00730 | chr12 | 53459012 | 53459012 | KRT80 | 27723759 |
| DE_00731 | chr12 | 52417086 | 52417086 | KRT80 | 27723759 |
| DE_00732 | chr12 | 52417086 | 52417086 | KRT80 | 27723759 |
| DE_00733 | chr4 | 1770844 | 1770844 | FGFR3 | 27723759 |
| DE_00734 | chr4 | 1762678 | 1762678 | FGFR3 | 27723759 |
| DE_00735 | chr17 | 61777324 | 61777324 | DCAF7 | 27723759 |
| DE_00736 | chr17 | 61513737 | 61513737 | DCAF7 | 27723759 |
| DE_00737 | chr17 | 61043674 | 61043674 | DCAF7 | 27723759 |
| DE_00738 | chr7 | 101930034 | 101930034 | CUX1 | 27723759 |
| DE_00739 | chr7 | 101362186 | 101362186 | CUX1 | 27723759 |
| DE_00740 | chr7 | 101362186 | 101362186 | CUX1 | 27723759 |
| DE_00741 | chr7 | 101589950 | 101589950 | CUX1 | 27723759 |
| DE_00742 | chr16 | 30753142 | 30753142 | SRCAP | 27723759 |
| DE_00743 | chr22 | 50627834 | 50627834 | TRABD | 27723759 |
| DE_00744 | chr22 | 50714820 | 50714820 | TRABD | 27723759 |
| DE_00745 | chr22 | 50639543 | 50639543 | TRABD | 27723759 |
| DE_00746 | chr22 | 50608627 | 50608627 | TRABD | 27723759 |
| DE_00747 | chr4 | 1805959 | 1805959 | FGFR3 | 27723759 |
| DE_00748 | chr20 | 46779797 | 46779797 | SULF2 | 27723759 |
| DE_00749 | chr20 | 46779797 | 46779797 | SULF2 | 27723759 |
| DE_00750 | chr20 | 46710095 | 46710095 | SULF2 | 27723759 |
| DE_00751 | chr20 | 47375521 | 47375521 | PREX1 | 27723759 |
| DE_00752 | chr20 | 47375521 | 47375521 | PREX1 | 27723759 |
| DE_00753 | chr20 | 47120374 | 47120374 | PREX1 | 27723759 |
| DE_00754 | chr20 | 61898173 | 61898173 | NKAIN4 | 27723759 |
| DE_00755 | chr17 | 81026079 | 81026079 | METRNL | 27723759 |
| DE_00756 | chr19 | 45981772 | 45981772 | PPM1N | 27723759 |
| DE_00757 | chr19 | 45981772 | 45981772 | PPM1N | 27723759 |
| DE_00758 | chr19 | 45972294 | 45972294 | PPM1N | 27723759 |
| DE_00759 | chr19 | 46319995 | 46319995 | PPM1N | 27723759 |
| DE_00760 | chr21 | 10816017 | 10816017 | TPTE | 27723759 |
| DE_00761 | chr21 | 10813552 | 10813552 | TPTE | 27723759 |
| DE_00762 | chr21 | 10832792 | 10832792 | TPTE | 27723759 |
| DE_00763 | chr21 | 10851202 | 10851202 | TPTE | 27723759 |
| DE_00764 | chr21 | 10820450 | 10820450 | TPTE | 27723759 |
| DE_00765 | chr21 | 10796504 | 10796504 | TPTE | 27723759 |
| DE_00766 | chr21 | 10849949 | 10849949 | TPTE | 27723759 |
| DE_00767 | chr21 | 10845180 | 10845180 | TPTE | 27723759 |
| DE_00768 | chr21 | 10780021 | 10780021 | TPTE | 27723759 |
| DE_00769 | chr21 | 10830716 | 10830716 | TPTE | 27723759 |
| DE_00770 | chr5 | 142782299 | 142782299 | NR3C1 | 27723759 |
| DE_00771 | chr5 | 141228722 | 141228722 | NR3C1 | 27723759 |
| DE_00772 | chr1 | 156470002 | 156470002 | TTC24 | 27723759 |
| DE_00773 | chr1 | 156561525 | 156561525 | TTC24 | 27723759 |
| DE_00774 | chr1 | 156645564 | 156645564 | TTC24 | 27723759 |
| DE_00775 | chr19 | 45954137 | 45954137 | PPM1N | 27723759 |
| DE_00776 | chr19 | 45954137 | 45954137 | PPM1N | 27723759 |
| DE_00777 | chr19 | 45394224 | 45394224 | PPM1N | 27723759 |
| DE_00778 | chr19 | 45942991 | 45942991 | PPM1N | 27723759 |
| DE_00779 | chr19 | 45349271 | 45349271 | PPM1N | 27723759 |
| DE_00780 | chr19 | 45811530 | 45811530 | PPM1N | 27723759 |
